# Supplementary material for: Paladin, a tyrosine phosphatase-like protein, is required for XA21-mediated immunity in rice
Source: Plant Commun. 2021 Jun 29;2(4):100215. doi: 10.1016/j.xplc.2021.100215 (PMC8299082; doi:10.1016/j.xplc.2021.100215)
Supplement: Document S1. Supplemental Figures 1–10 and supplemental Table 1 [file mmc1.pdf]

Plant Communications, Volume *2*

Supplemental information

Paladin, a tyrosine phosphatase-like protein, is required for *XA21*-mediated immunity in rice

Tsung-Chi Chen, Mawsheng Chern, Michael Steinwand, Deling Ruan, Yu Wang, Arkin Isharani, and Pamela Ronald

***Paladin* (*PALD*), a tyrosine phosphatase-like protein, is required for *XA21*-mediated immunity in rice**

Tsung-Chi Chen*, Mawsheng Chern*, Michael Steinwand, Deling Ruan, Yu Wang, Arkin Isharani, and Pamela Ronald

Abstract

XA21 encodes a rice immune receptor that confers robust resistance to most strains of the Gram-negative bacterium *Xanthomonas oryzae* pv. *oryzae* (*Xoo*). *XA21*-mediated immunity is triggered by recognition of a small protein called RaxX-sY (required for activation of *XA21*-mediated immunity X, tyrosine-sulfated) secreted by *Xoo.* To identify components regulating *XA21*-mediated immunity, we generated and screened a mutant population of fast neutron mutagenized rice expressing *Ubi:Myc-XA21* for those susceptible to *Xoo*. Here, we report the characterization of one of these rice mutants, named *sxi2* (*suppressor of XA21-mediated immunity-2*). Whole genome sequencing reveals that *sxi2* carries a deletion of the *PALADIN* (*PALD*) gene that encodes a protein with three putative protein tyrosine phosphatase-like domains (PTP-A, B, and C). Expression of *PALD* in the *sxi2* genetic background is sufficient to complement the susceptible phenotype, and requires the catalytic cysteine of the PTP-A active site to restore resistance. PALD co-immunoprecipitates with full-length XA21. XA21 protein levels are positively regulated by the presence of the *PALD* transgene. We find that *sxi2* retains many hallmarks of XA21-mediated immunity similar to wild type. These results demonstrate a function for PALD, a previously uncharacterized class of phosphatase, in rice innate immunity, and the requirement of the conserved cysteine in the PTP-A domain for PALD function.

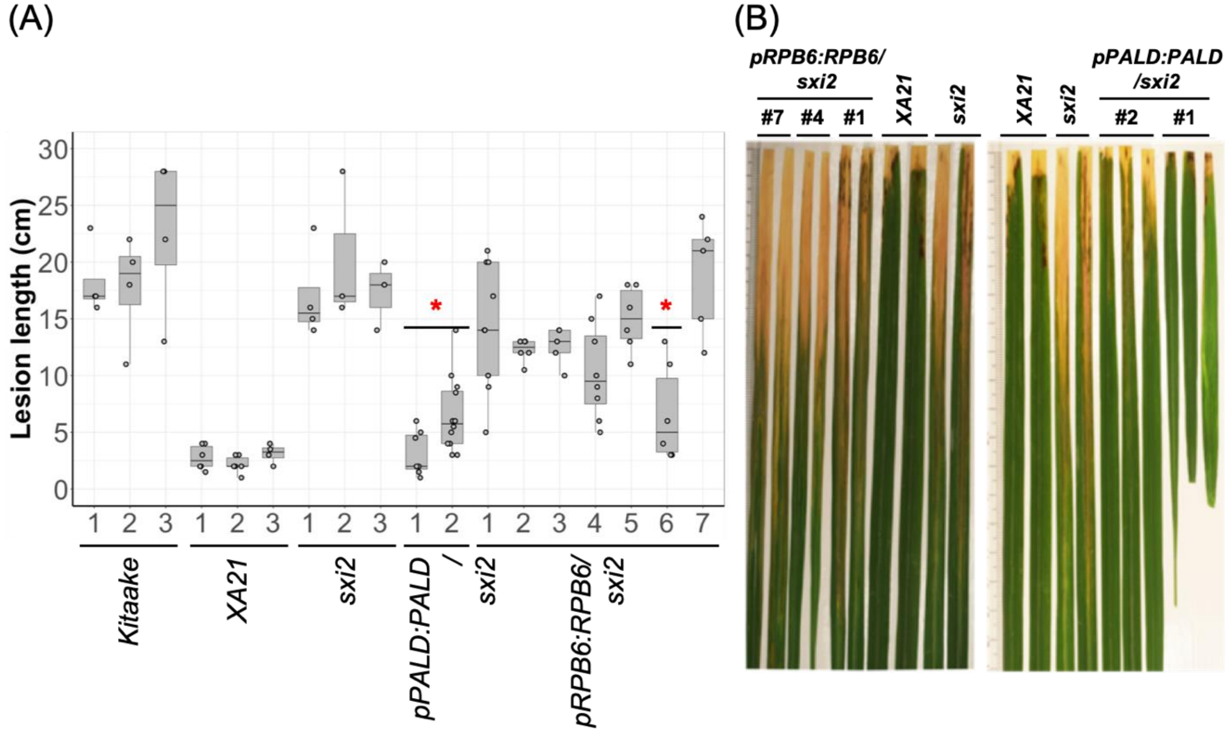

Supplemental Figure 1. The PALD gene complements the *sxi2* susceptible phenotype in T0 plants. **(A)** Lesion lengths of two *pPALD:PALD/sxi2* and seven *pRPB6:RPB6/sxi2* T0 complementation lines. Three independent transgenic lines were inoculated by scissor clipping with *Xoo* strain PXO99 along with the control plants. Whisker plot and represent the medium, upper and lower quartiles of lesion measurements (cm) on Kitaake, XA21, *sxi2*, *pPALD:PALD/sxi2* complementation lines, and *pRPB6::RPB6/sxi2* complementation lines 14 d post-inoculation. **(B)** Image of the inoculation results of **(A).** *P < 0.05 compared with *sxi2* using Dunnett's test.

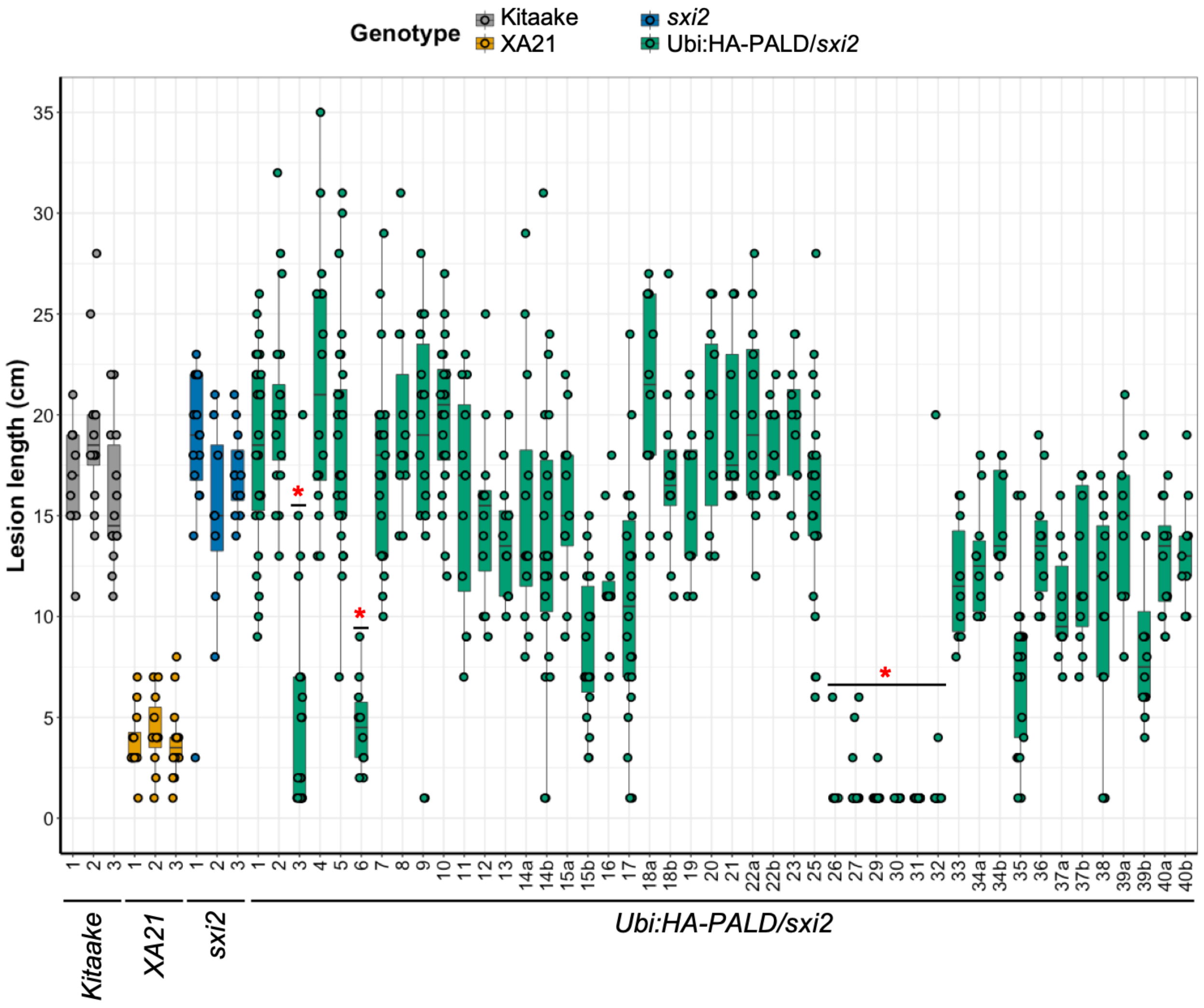

Supplemental Figure 2. Lesion lengths of *Ubi:HA-PALD/sxi2* T0 plants after *Xoo* inoculation. To test if the overexpression of PALD rescues the susceptible phenotypes of *sxi2*, 38 independent T0 plants (*Ubi:HA-PALD/sxi2*) were inoculated with *Xoo* strain PXO99. Lesion lengths were measured two weeks after the inoculation. Rice seeds from the tillers showing resistant phenotypes (with shorter than 8 cm of lesion length) were harvested and inoculated again in the T1 generation to test if the resistant phenotypes are heritable. The inoculation results of T1 plants derived from T0 lines #3, #25, #26, #29, #30, #31, and #32 are shown in Figure 2B. *$P < 0.05$ compared with *sxi2* using Dunnett's test.

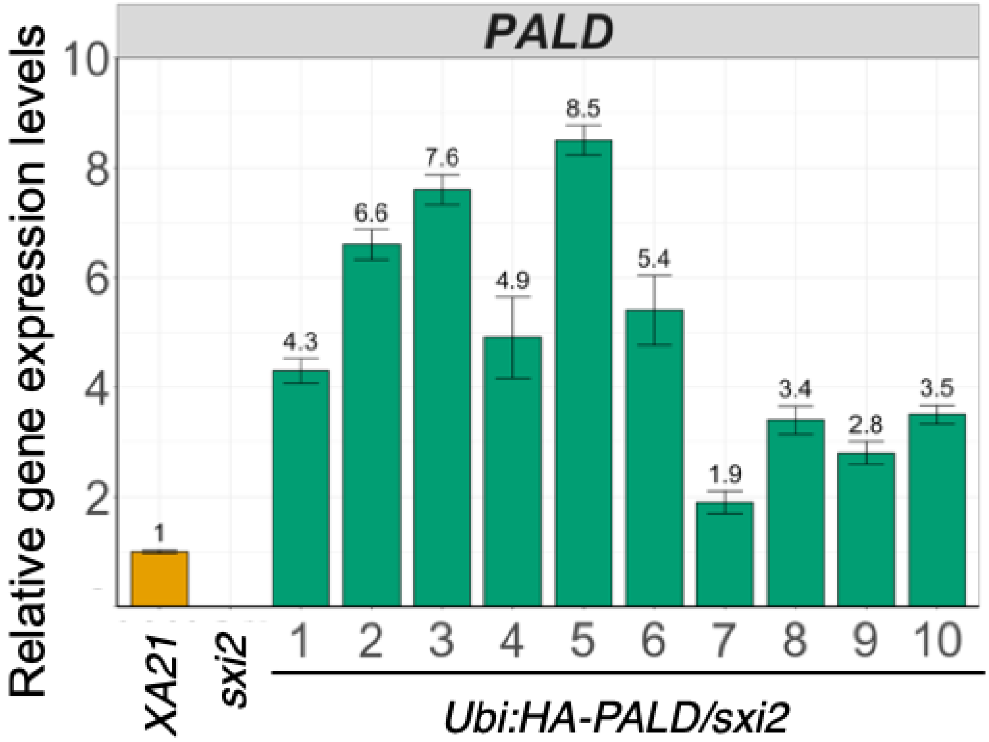

Supplemental Figure 3. Quantitative RT-PCR analysis of *PALD* expression levels in the leaves of 10 independent T0 populations of Ubi:HA-PALD/sxi2 transgenic plants. Quantitative RT-PCR analysis of *PALD* expression levels in the leaves of T0 populations of 10 independent Ubi:HA-PALD/sxi2 transgenic plants (from line #1 to #10). The expression level of *PALD* is normalized to the XA21 control. PALD expression levels in all Ubi:HA-PALD/sxi2 plants are significantly higher than XA21 ($P < 0.05$, Student's t-test). This experiment was done once with three biological replicates.

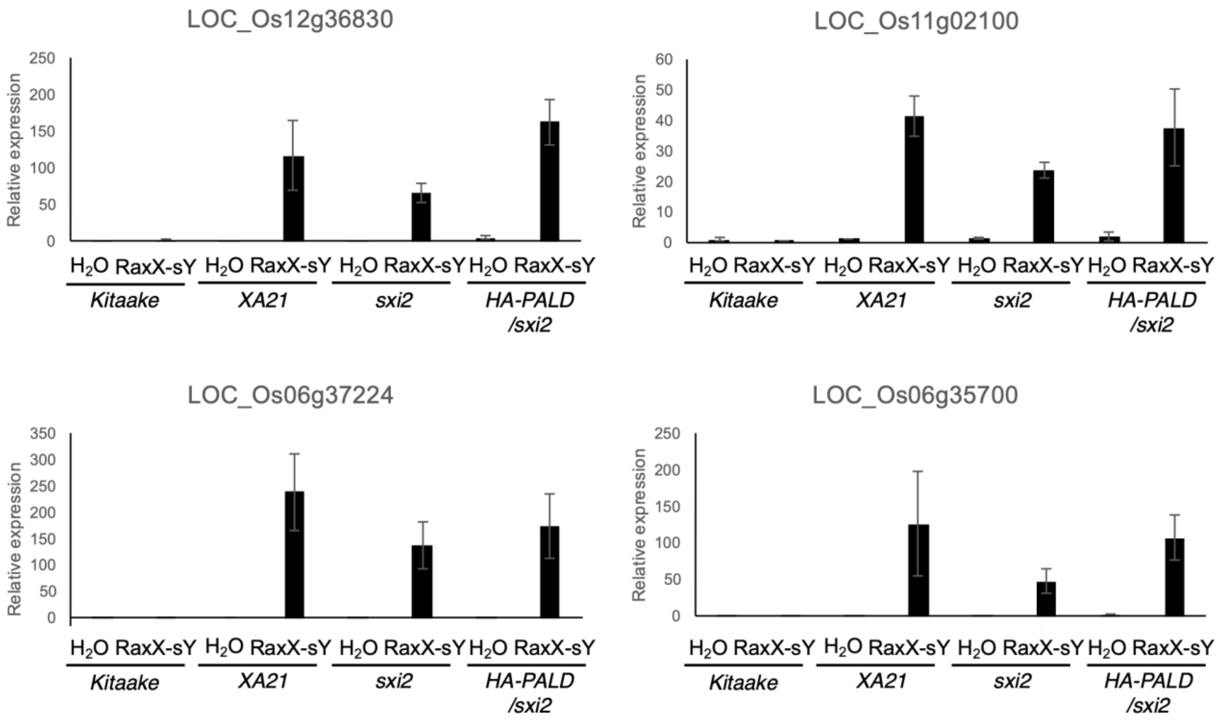

Supplemental Figure 4. Four defense marker genes are up-regulated in the three XA21-carrying genotypes. The relative expression levels of four defense marker genes after 8 hours of RaxX-sY or H_2O treatment. The expression levels of these genes are normalized to the Kitaake-H_2O samples. Bars and error bars represent the mean ± SD of relative expression levels in Kitaake, XA21, *sxi2*, *HA-PALD/sxi2* transgenic lines 8 hours after treatment (n = 3).

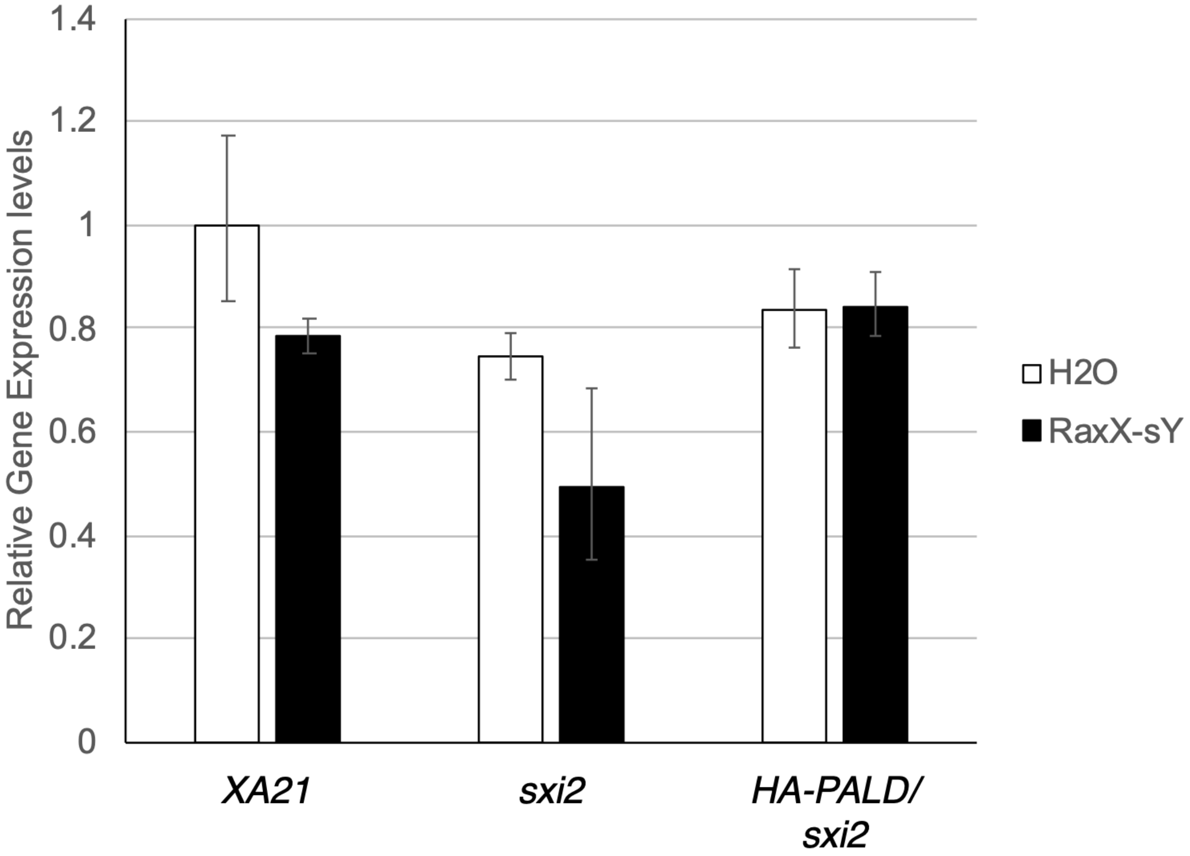

Supplemental Figure 5. qRT-PCR analysis of XA21 expression levels among the three XA21-carrying genotypes. Transcript levels of *XA21* in leaves of *XA21*, *sxi2*, and *HA-PALD/sxi2* rice plants treated with H_2O (mock) or RaxX21-sY (500 nM, n = 3) for 8 hours. All data points depict means ± SE. These experiments were repeated three times with similar results.

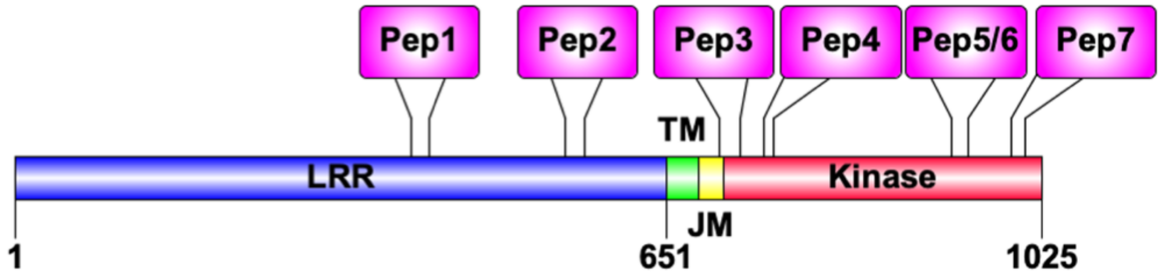

Peptide	Sequence	Number of Hits_rep1	Number of Hits_rep2	Number of Hits_rep3
1	DIGNLIGLQHLYLcNNNFR	1		
2	YLYLQNNLLSGSIPSALGQLK	1		
3	ATDGFAPTNLLGSGSFGSVYK	2		
4	SFTAEcEALR	1	1	
5	QYVELGLHGR		1	
6	VTDVVDTK	1		
7	TPTGDIIDELNAIK	1	2	1

Supplemental Figure 6. Liquid chromatography-mass spectrometry analysis identifies XA21 as an *in vivo* interactor of PALD. Co-immunoprecipitated HA-PALD samples were subjected to analysis with LC-MS/MS to identify putative interactors. Seven peptides of the XA21 receptor were identified, matched to the LRR and kinase protein domains. In three replicate samples from separate experiments, peptides matched to the kinase domain recur most often.

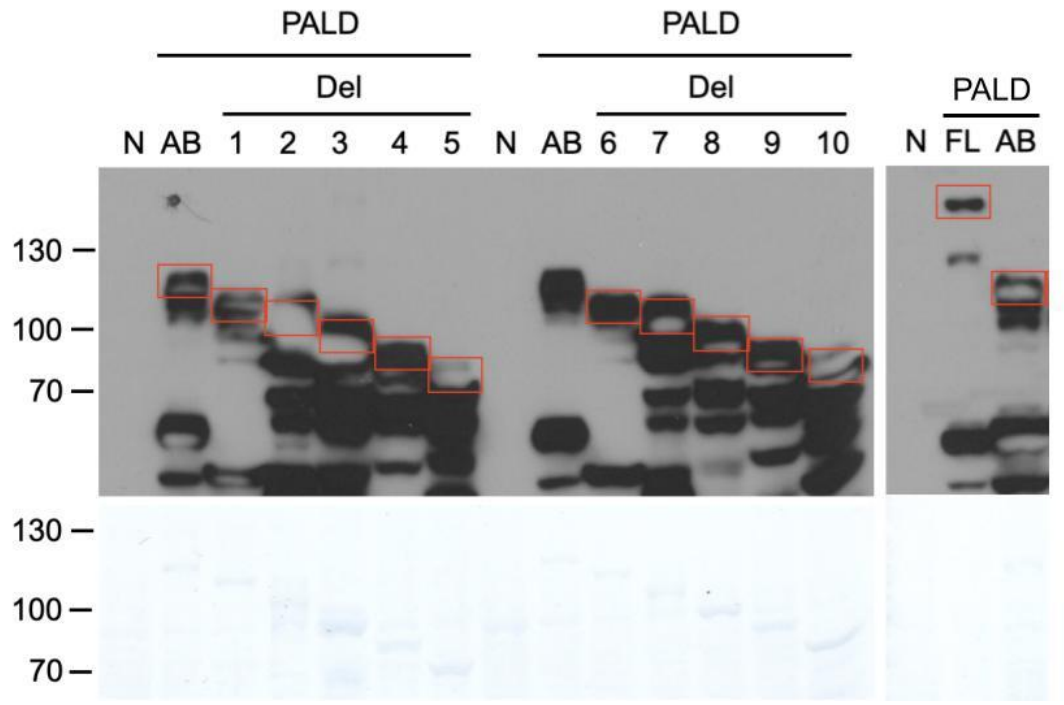

Supplemental Figure 7. Western blot analysis of PALD proteins expressed in yeast. Upper panel: We performed Western blot analysis to measure the expression of different truncation forms of PALD-HA proteins used in the Yeast two-hybrid assays (Figure 6). Yeast samples carrying different truncation forms of PALD were lysed for protein extraction. Protein samples were loaded and separated on 8% SDS-PAGE followed by Western blot analysis. The red squares are the estimated size of the specified PALD-HA. Lower panel: Coomassie blue staining was used as loading control. N: negative control, empty vector; AB: PALD-AB; FL: PALD-FL; 1 to 10: Del-1 to Del-10.

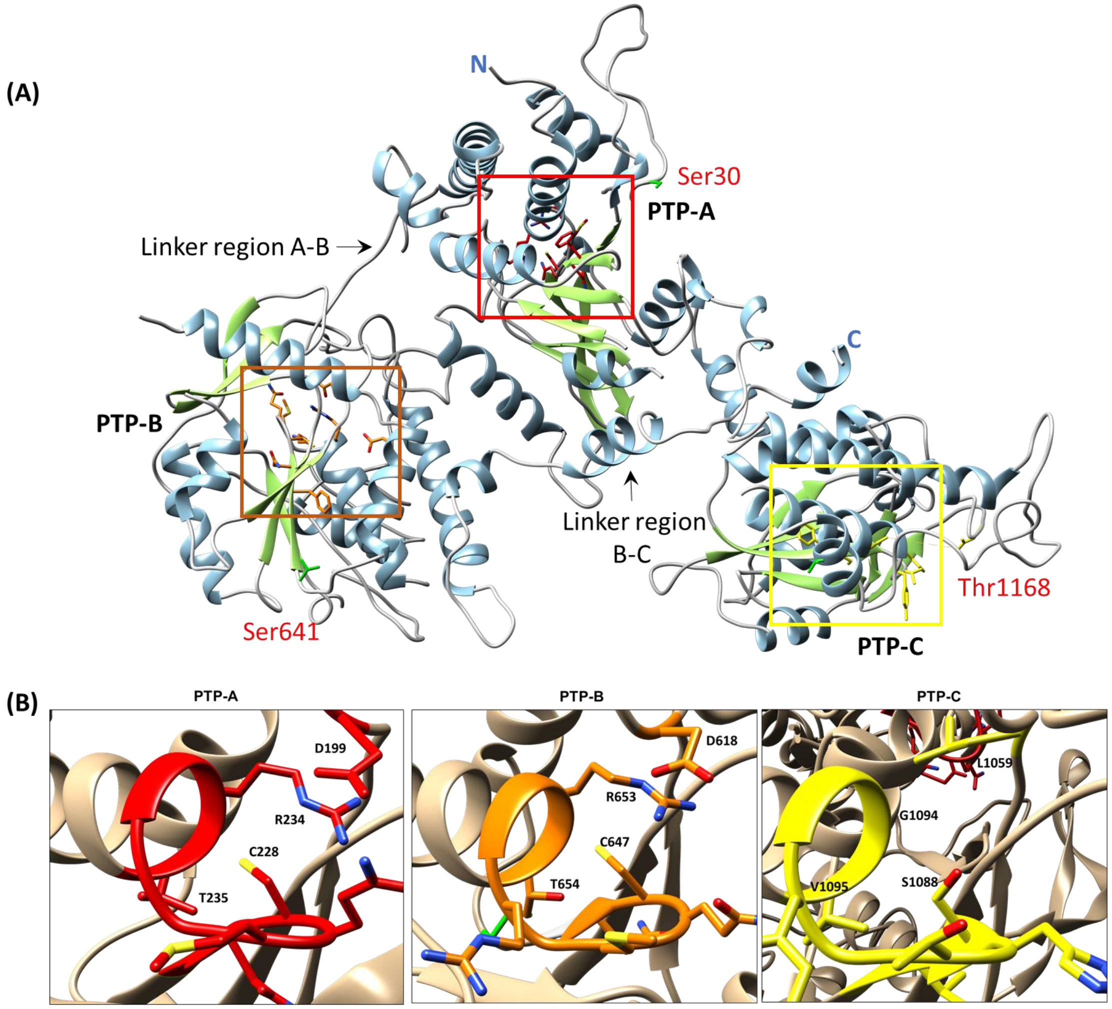

(C)

Peptides identified in MS	H_2O_1h	RaxX-sY_1h
**S**^{30}DHFPGCQNK	4	2
S^{30p}DHFPGCQNK	2	2
D**T**^{641}AFVFN**C**QMGR	0	0
D***T***^{641p}AFVFN**C**QMGR	1	1
EEVD**T**^{1168}VIDR	6	6
KEEVD**T**^{1168}VIDR	2	2
CKEEVD***T***^{1168p}VIDR	1	0

Supplemental Figure 8. Structural homology model and phosphorylated amino acid residues of PALD (A) Structural prediction of PALD crystal structure was performed using RaptorX homology modeling software (Template 4WU3). PTP domains with active site residues shown are boxed, three phosphorylated residues (Ser^{30}, Thr^{641}, and Thr^{1168} sidechains in green, labels in red)

and putative linker regions. **(B)** Model of the active site of PTP-A, B and C. PTP-A and PTP-B possess a catalytic cysteine (C228, 647), arginine (R234, 653) threonine (T235, 654) on the p-loop, and aspartate (D199, 618) on the general acid loop, while these residues are absent in PTP-C. **(C)** Three phosphopeptides were detected on HA-PALD via mass spectrometry. Three amino acids (S30, T641, and T1168) of PALD are phosphorylated *in vivo* in the absence of RaxX21-sY treatment. No significant changes were detected after RaxX21-sY peptide treatment as compared with the water control.

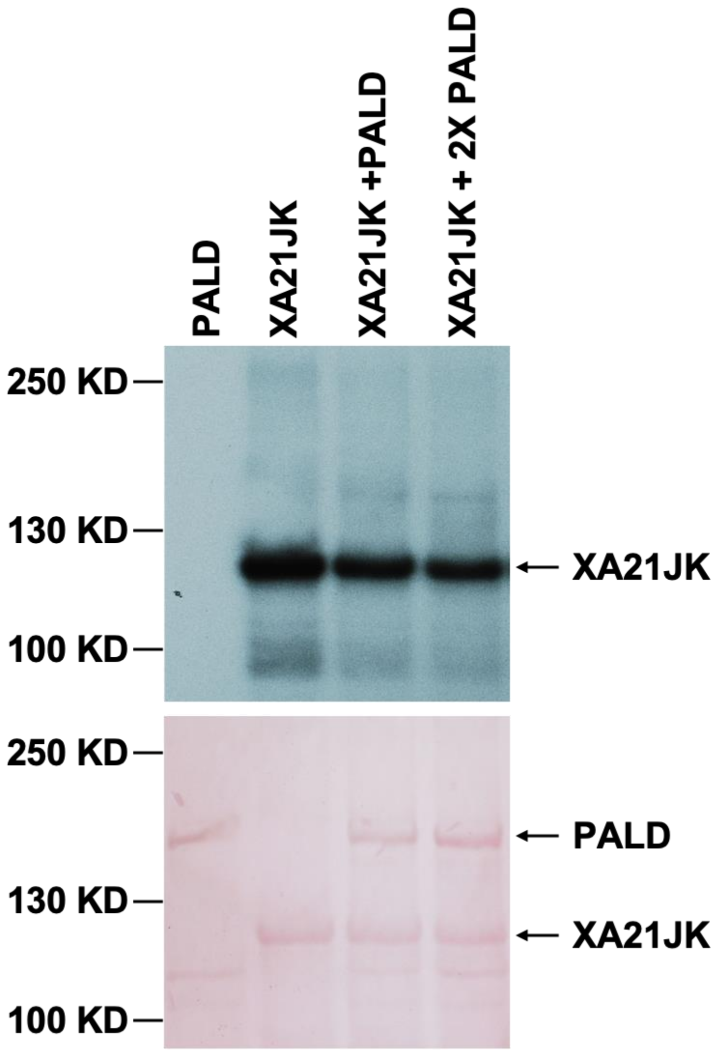

Supplemental Figure 9. XA21 does not phosphorylate PALD *in vitro*.

This *in vitro* kinase assay was performed by incubating recombinant His-Nus-XA21 in the presence or absence of His-PALD using [^{32}P]-γ-ATP. Proteins were separated by SDS-PAGE and analyzed by autoradiography. Lower panel, ponceau staining of the same PVDF membrane shown in upper panel as a protein loading control. This experiment was repeated twice with similar results.

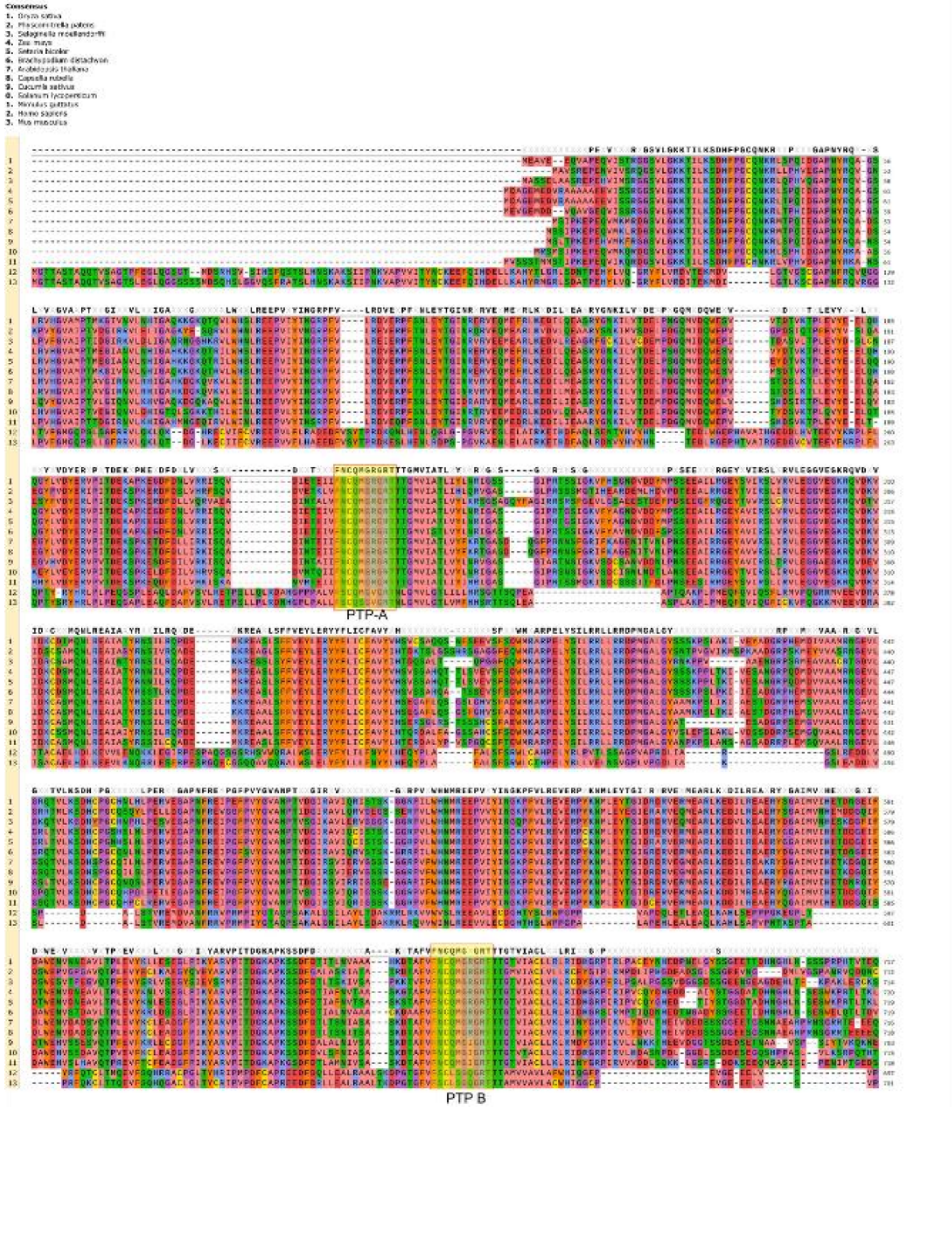
Consensus
1. Oryza sativa
2. Physcomitrella patens
3. Selaginella moellendorffii
4. Zea mays
5. Setaria bicolor
6. Brachypodium distachyon
7. Arabidopsis thaliana
8. Capsella rubella
9. Cucumis sativus
0. Solanum lycopersicum
1. Mimulus guttatus
2. Homo sapiens
3. Mus musculus
PTP-A
PTP B

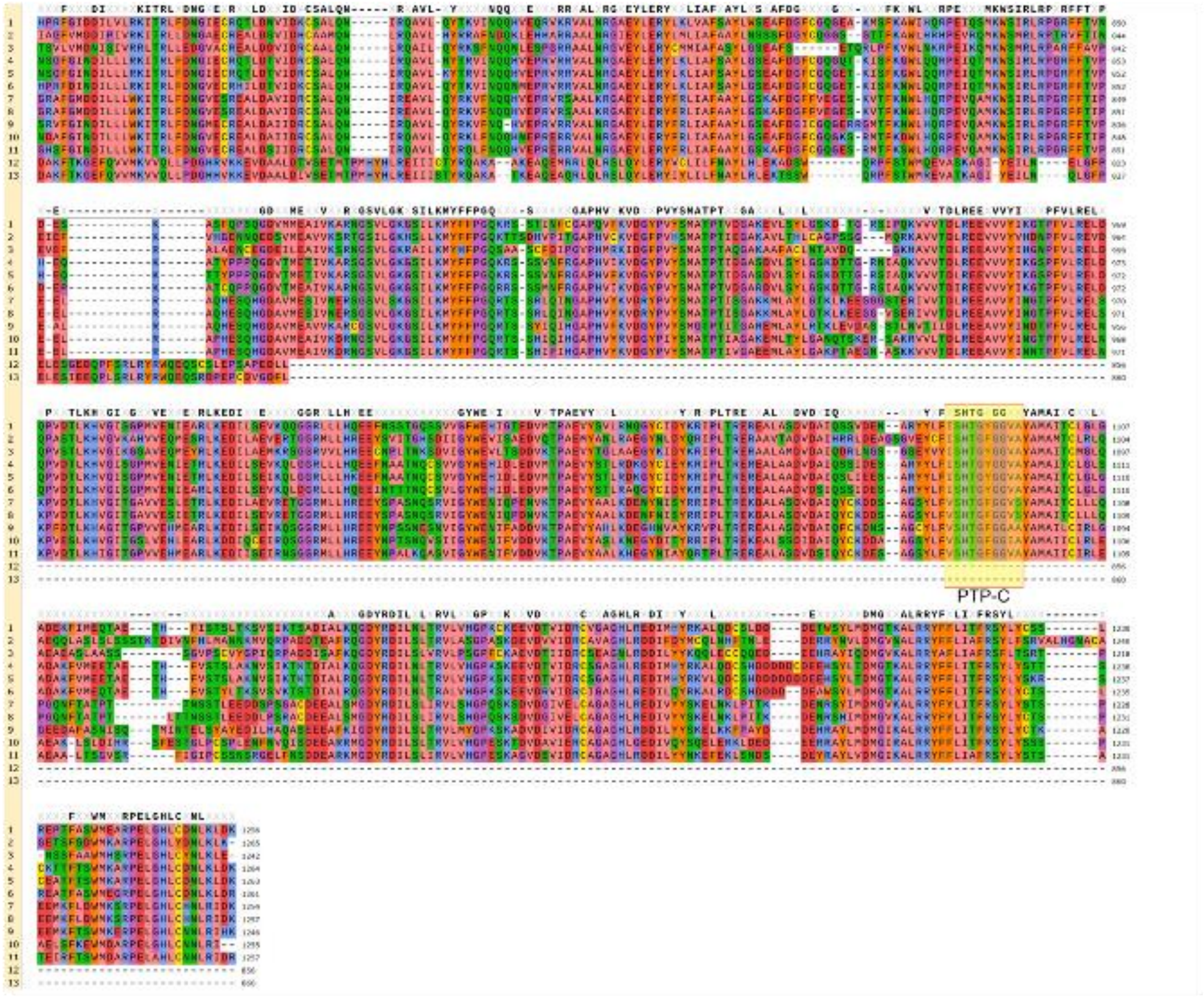

Supplemental Figure 10. Sequence alignment of select PALD proteins from plant and animals

Supplemental information on HA-PALD lines:

Among the 38 Ubi:HA-PALD/sxi2 T0 transgenic plants, 13 plants (line-#3, #6, #14b, #15b, #17, #25, #26, #27, #29, #30, #31, #32, and #35) displayed resistance to *Xoo* in at least one rice leaf, while all the other lines were susceptible (Supp. Figure 2). We harvested the seeds from the resistant tillers of the 13 plants and repeated the inoculation in the derived T1 population. We found that progeny of *Ubi:HA-PALD/sxi2*-#3, #25, and #26 are the only lines that displayed resistant phenotypes similar to the *XA21* control plants (Figure 2B). We then assessed their *PALD* RNA levels by reverse transcription quantitative PCR (RT-qPCR) analysis (Supp. Figure 3) and their protein levels by immunoblotting (Figure 2C) on the T0 populations. The RT-qPCR results showed that *PALD* transcript levels in the ten Ubi:HA-PALD/sxi2 transgenic lines (from line #1 to #10) are at least 2-fold higher than XA21 plants, suggesting that the *PALD* gene is overexpressed in these transgenic plants (Supp. Figure 3). However, immunoblot results showed that Ubi:HA-PALD/sxi2-#3, -#25, and -#26, the only resistant lines, express easily detectable levels of the HA-PALD fusion protein among all transgenic lines, whereas the remaining 35 Ubi:HA-PALD/sxi2 lines express lower or undetectable levels of the PALD protein and are susceptible to *Xoo* strain PXO99 (Figure 2B, 2C, and 2D). These results show that detectable HA-PALD protein is correlated with the *Xoo* resistant phenotype, suggesting that the PALD protein is required for *XA21*-mediated immunity.

Supplemental table S1. Primers used in this study.

Gene	Locus ID	Use	Forward primer	Reverse Primer
Actin	LOC_Os03g50885	qRT-PCR	ACAGGTATTGTGTTGGACTCTGG	AGTAACCACGCTCCGTCAGG
PALD	LOC_Os03g38970	qRT-PCR	ATCGCCAGGCGGGTTCTTTA	CGGCTCCTCGCGAAGACTAT
UBQ	LOC_Os06g46770	qRT-PCR	GTGGCCAGTAAGTCCTCAGC	ACAATGAAACGGGACACGAC
PALD	LOC_Os03g38970	Genotyping (primer 1)	AAGCGGAACGCTATAGTGGG	AAGGTGACCATTGTGATCCGTTGTT
	LOC_Os08g29590	Genotyping (Primer 2)	CACCGAATTCATGAGGATGGACAGGAATGAGGA	GTCAAGGCATGAGCGGTGAACA
	LOC_Os09g20830	Genotyping (Primer 3)	CTCTCGCTCGTGTTCGTCCTC	AGCTCCACACCTCTCTTGACCA
