# Supplementary material for: Paladin, a tyrosine phosphatase-like protein, is required for XA21-mediated immunity in rice
Source: Plant Commun. 2021 Jun 29;2(4):100215. doi: 10.1016/j.xplc.2021.100215 (PMC8299082; doi:10.1016/j.xplc.2021.100215)
Supplement: Document S2. Article plus supplemental information [file mmc2.pdf]

Plant Communications
Research Article

CellPress
Partner Journal

Paladin, a tyrosine phosphatase-like protein, is required for *XA21*-mediated immunity in rice

Tsung-Chi Chen[1,*], Mawsheng Chern[1,*], Michael Steinwand[*], Deling Ruan[*], Yu Wang[*], Arkin Isharani[*] and Pamela Ronald[*]

Department of Plant Pathology and the Genome Center, University of California, Davis, CA 95616, USA

[1]These authors contributed equally to this article.

***Correspondence: Tsung-Chi Chen (tcch@ucdavis.edu), Mawsheng Chern (mschern@ucdavis.edu), Michael Steinwand (masteinwand@ucdavis.edu), Deling Ruan (dlruan@ucdavis.edu), Yu Wang (yuwwang@163.com), Arkin Isharani (acisharani@ucdavis.edu), Pamela Ronald (pcronald@ucdavis.edu)**

https://doi.org/10.1016/j.xplc.2021.100215

ABSTRACT

***XA21* encodes a rice immune receptor that confers robust resistance to most strains of the Gram-negative bacterium *Xanthomonas oryzae* pv. *oryzae* (*Xoo*). *XA21*-mediated immunity is triggered by recognition of a small protein called RaxX-sY (required for activation of *XA21*-mediated immunity X, tyrosine-sulfated) secreted by *Xoo.* To identify components regulating *XA21*-mediated immunity, we generated and screened a mutant population of fast-neutron-mutagenized rice expressing *Ubi:Myc-XA21* for those susceptible to *Xoo*. Here, we report the characterization of one of these rice mutants, named *sxi2* (*suppressor of XA21-mediated immunity-2*). Whole-genome sequencing revealed that *sxi2* carries a deletion of the *PALADIN* (*PALD*) gene encoding a protein with three putative protein tyrosine phosphatase-like domains (PTP-A, -B, and -C). Expression of *PALD* in the *sxi2* genetic background was sufficient to complement the susceptible phenotype, which requires the catalytic cysteine of the PTP-A active site to restore resistance. PALD co-immunoprecipitated with the full-length XA21 protein, whose levels are positively regulated by the presence of the *PALD* transgene. Furthermore, we foundd that *sxi2* retains many hallmarks of *XA21*-mediated immunity, similar to the wild type. These results reveal that PALD, a previously uncharacterized class of phosphatase, functions in rice innate immunity, and suggest that the conserved cysteine in the PTP-A domain of PALD is required for its immune function.**

Key words: XA21, rice bacterial blight disease, *Xanthomonas oryzae* pv. *oryzae*, fast-neutron mutagenesis, PALD

Chen T.-C., Chern M., Steinwand M., Ruan D., Wang Y., Isharani A., and Ronald P. (2021). Paladin, a tyrosine phosphatase-like protein, is required for *XA21*-mediated immunity in rice. Plant Comm. **2**, 100215.

INTRODUCTION

Plants and animals utilize immune receptors to recognize and respond to microbes. Plants deploy immune receptors at the cell surface that detect conserved immunogens produced by pathogens. These immune receptors may form protein complexes with co-receptors and receptor-like cytoplasmic kinases upon recognition of cognate molecular pattern ligands to activate plant defense responses. Research in rice, wheat, *Arabidopsis*, and tomato suggest that the molecular mechanisms of immune signaling downstream of these immune receptors are highly conserved (Boutrot and Zipfel, 2017). Understanding these underlying signaling pathways advances our knowledge of the mechanisms of plant immunity and can guide efforts to engineer enhanced resistance in agricultural crops.

In *Arabidopsis* and rice, recognition of cognate ligands by immune receptors triggers rapid immune responses across several time frames. Within minutes, bursts of reactive oxygen species (ROS) production and a cascade of mitogen-activated protein kinases phosphorylation convey the immune signal generated from membrane-bound immune receptors to nuclear components (Couto and Zipfel, 2016; Pruitt et al., 2015). Late immune responses, triggered hours after ligand perception, include the production of ethylene and accumulation of pathogenesis-related protein gene transcripts (Kim et al., 2016; Pruitt et al., 2015). These immune responses contribute to disease resistance in many plant–pathogen interactions (Chen et al., 2014; Macho and Zipfel, 2014; Pruitt et al., 2015).

Rice plants carrying the XA21 immune receptor exhibit robust resistance to strains of the bacterial pathogen *Xanthomonas oryzae* pv. *oryzae* (*Xoo*) that secrete a small tyrosine-sulfated peptide

Published by the Plant Communications Shanghai Editorial Office in association with Cell Press, an imprint of Elsevier Inc., on behalf of CSPB and CEMPS, CAS.

This is an open access article under the CC BY license (http://creativecommons.org/licenses/by/4.0/).

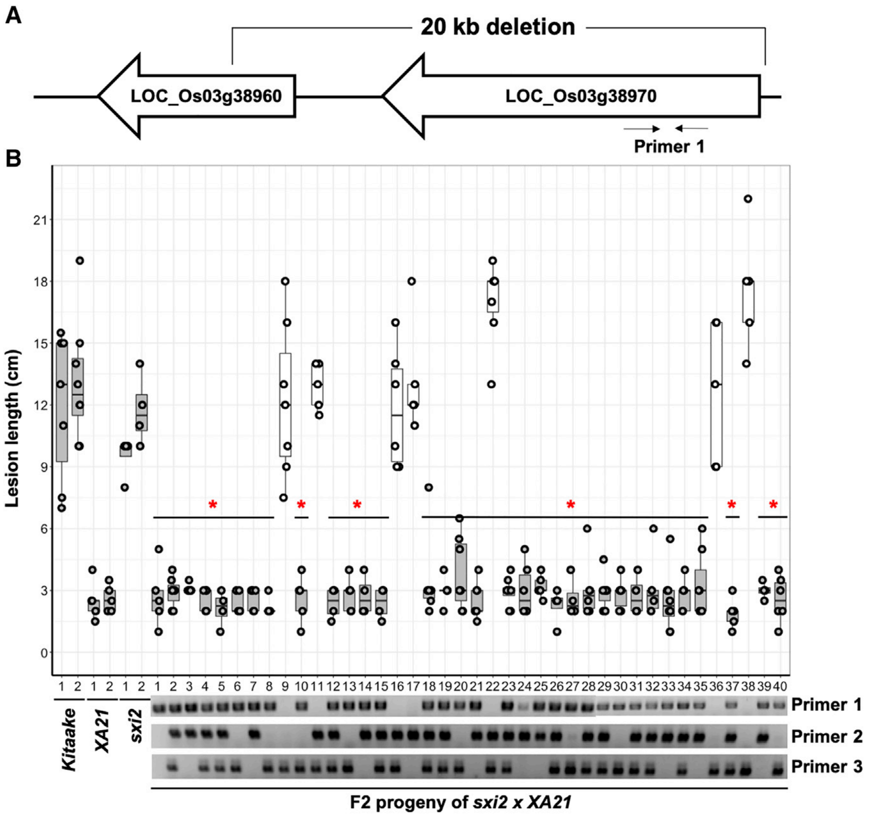

Figure 1. A chromosome 3 deletion co-segregates with the *sxi2* susceptible phenotype in an F2 population

(A) Diagram showing the two genes localized in the chromosome 3 20-kb deletion in the3 *sxi2* mutant genome. The sequence targeted by the primer 1 pairs are indicated.

(B) Lesion length and PCR genotyping results of the F2 progeny (n = 4–7), derived from a cross of *sxi2* with the XA21 parental genotype, inoculated with *Xoo*. Controls plants include Kitaake, XA21, and *sxi2.* Lesion lengths were measured 14 days post-inoculation. Each dot represents a single leaf inoculation. Lower panel: genotyping results of three different sets of primers targeting three different mutations on chromosome 3 of each F2 individual. Primer 1 targets a deletion region in chromosome 3 of *sxi2* (see **A**). Primer pairs 2 and 3 target two different single-base pair substitutions nearby the deletion region in LOC_Os03g10990. The absence of a PCR product indicates a homozygous deletion or mutation; the presence of a PCR product indicates that the plant carries at least one copy of the wild-type allele. Primer sequences are listed in Supplemental Table 1. *$P < 0.05$ compared with *sxi2* using Dunnett's test.

called RaxX (RaxX-sY) (required for activation of *XA21*-mediated immunity X, tyrosine-sulfated) (Luu et al., 2019; Pruitt et al., 2015). XA21 is a receptor-like kinase consisting of an extracellular leucine-rich repeat (LRR) domain, a transmembrane region, a juxtamembrane motif, and an intracellular non-arginine-aspartate (non-RD) kinase domain (Park et al., 2012). Transgenic rice plants overexpressing an N-terminal Myc-tagged XA21 display robust resistance to *Xoo* strain PXO99; in contrast, wild-type rice that lacks *XA21* is susceptible to *Xoo* strain PXO99, reflected by the formation of long disease lesions from the point of inoculation along the leaf (Park et al., 2010a). The XA21 LRR domain directly binds a synthetic, 21 amino acid, tyrosine-sulfated RaxX (RaxX21-sY) with high affinity (K_d = 16 nM) (Luu et al., 2019). This binding triggers a ROS burst and ethylene production, and induces expression of defense marker genes, such as *PR10b* (LOC_Os12g36850) and LOC_Os04g10010 in *XA21* plants (Luu et al., 2019; Pruitt et al., 2015). Despite these advances, the detailed molecular and physiological mechanisms underlying *XA21* resistance have yet to be fully determined.

In this study, we characterized a rice mutant called *sxi2* (*suppressor of XA21-mediated immunity 2*) that was identified in a forward genetic screen by direct inoculation with PXO99*.* We identified a 20-kb deletion in chromosome 3 of *sxi2* that co-segregates with the susceptible phenotype and found that genetic complementation with one of the deleted genes, *PALADIN* (*PALD*), rescues the susceptible phenotype of *sxi2*. *PALD* encodes a protein that carries three putative protein tyrosine phosphatase (PTP) domains. We further found that the first PTP domain is critical for PALD to complement the *sxi2* mutant. PALD interacts with XA21 in rice protein co-immunoprecipitation and in yeast two-hybrid assays. In contrast, the ROS burst and expression of defense marker genes, such as *LOC_Os12g36830* and *LOC_Os06g37224*, are not affected in *sxi2*. Together, these results demonstrate a role for the phosphatase PALD, a previously undescribed class of PTPs, in *XA21*-mediated immunity.

RESULTS

The susceptible phenotype of *sxi2* co-segregates with a deletion locus in chromosome 3

To study the molecular mechanism underlying *XA21*-mediated immunity, we carried out a forward genetic screen of the rice variety Kitaake expressing the *XA21* gene to isolate *Xoo*-susceptible mutants. This Kitaake transgenic line expresses *Ubi:Myc-XA21* (called the *XA21* line in this manuscript) and displays robust resistance to *Xoo* strain PXO99 (Pruitt et al., 2015). This *XA21* line was mutagenized by fast-neutron treatment, generating a population containing over 6000 M1 lines (Li et al., 2017). Approximately 21 000 M2 lines (derived from 3000 M1) were inoculated with *Xoo*, and 10 mutants that carry compromised *XA21*-mediated immunity were isolated and named suppressors of *XA21*-mediated immunity (*sxi*). Here, we focus on the mutant *sxi2*.

To identify the underlying mutation associated with the susceptibility phenotype, *sxi2* was backcrossed with its *XA21* parent to generate a segregating F2 population. Inoculation of 40 F2 plants revealed that 7 progenies are fully susceptible to *Xoo* and 33 are resistant (Figure 1), suggesting that a recessive mutation in *sxi2* plants is causing the susceptible phenotype. To identify the responsible mutation, we sequenced the genome of *sxi2* and

identified three large deletions distributed on chromosomes 1, 3, and 8, respectively. Using 3 pairs of primers each targeting one of these 3 deletions to PCR genotype the *sxi2* mutant in 40 segregating F2 progeny derived from a backcross with the Ubi:Xa21 parent, we found that only the primer pair targeting the 20-kb deletion on chromosome 3 of *sxi2* (shown as primer 1 in Figure 1B) co-segregated with the susceptible phenotype. This result suggests that one or more of the genes deleted in this region is required for *XA21*-mediated immunity. In addition to this deletion, two non-synonymous single-base substitution mutations are found nearby LOC_Os03g10990. We also PCR genotyped these two single-base substitutions in the same F2 population, and the result confirmed that the susceptible phenotype is totally linked to the 20-kb deleted region but not to the two single-base substitutions (Figure 1B).

Complementary expression of *PALD* rescues the susceptible phenotype of *sxi2*

The 20-kb deleted region contains two predicted protein-coding genes: *Paladin* (*PALD*, LOC_Os03g38970) and *DNA-directed RNA Polymerase Subunit 6* (*RPB6*, LOC_Os03g38960). *PALD* encodes a putative PTP with unknown function that carries three predicted PTP domains (PTP-A, amino acids [aa] 91–245; PTP-B, aa 509–665; PTP-C, aa 948–1104) that share high protein sequence similarity to pfam14566, a PTP-like phytase protein family (Alonso and Pulido, 2016). *RPB6* encodes a subunit of RNA polymerase that catalyzes transcription. To test whether *PALD* or *RPB6* can restore *XA21*-mediated resistance in *sxi2* plants, *RPB6* and *PALD*, each including their respective promoter and coding region, were separately introduced into the *sxi2* mutant via *Agrobacterium*-mediated transformation. We obtained two independent T0 lines for *PALD* (pPALD:PALD/sxi2) and seven T0 lines for *RPB6* (pRPB6:RPB6/sxi2). Inoculation experiments indicate that the two *PALD* lines (*pPALD:PALD/sxi2* nos. 1 and 2) were resistant to *Xoo* strain PXO99, whereas six out of seven complementation lines carrying *RPB6* (from *pRPB6:RPB6/sxi2* nos. 1–6) displayed susceptible phenotypes. One *RPB6* T0 line showed partial resistance (Supplemental Figure 1). In the subsequent T1 generation, only transgenic lines carrying *PALD* (*pPALD:PALD/sxi2* nos. 1 and 2) were resistant to *Xoo*, and the resistance co-segregated with the transgene (Figure 2A). These results suggest that *PALD* but not *RPB6* can complement the *XA21*-mediated immune response in the *sxi2* mutant.

We also generated transgenic plants that express the *PALD* coding sequence tagged with a hemagglutinin (HA) epitope at its N terminus under control of the maize ubiquitin promoter (*Ubi:HA-PALD*) in the *sxi2* background. The Myc-XA21 expression construct used in these experiments is driven by the maize *ubiquitin-1* promoter, which extends XA21 protein expression to all cell types in the rice leaf. We obtained 38 independent T0 *Ubi:HA-PALD/sxi2* lines, which were assessed for *PALD* RNA and protein levels, and resistance to *Xoo*. Only three T0 lines (nos. 3, 25, and 26) gave rise to T1 progeny that express sufficient HA-PALD protein to confer heritable resistance to *Xoo* (Figure 2C and Supplemental Figure 2). We found that detectable HA-PALD protein did not correlate with *HA-PALD* RNA quantity, because many T0 lines that showed undetectable protein levels displayed similar *PALD* transcript levels as line no. 3 (Supplemental Figure 3). Despite the low frequency of detectable HA-PALD protein, the heritability of the complemented phenotype in line nos. 3, 25, and 26 further supports that the PALD protein is required for *XA21*-mediated immunity and sufficient to complement *sxi2*.

ROS production and expression defense marker genes are not altered in *sxi2* upon recognition of RaxX21-sY by XA21

In our previous work, we observed that application of RaxX21-sY synthetic peptide to *XA21* rice leaves triggers hallmarks of the innate immune response, including ROS production via plasma membrane RBOH proteins and induction of several defense marker genes (Pruitt et al., 2015). Using reagents that convert ROS to luminescence, we observed clear bursts of ROS production in *XA21*, *sxi2*, and *HA-PALD/sxi2* plants that were similar in both amplitude over time and cumulative chemiluminescence (Figure 3). Chemiluminescence was not significantly elevated in water or non-sulfated RaxX21 treatments, nor in Kitaake that lack *XA21*. RNA expression analysis of leaf clippings treated with RaxX21-sY for 8 h also does not show a change in expression of established marker genes in *sxi2* relative to *XA21* (Supplemental Figure 4). These results demonstrate that ROS bursts are specifically elicited by RaxX21-sY, and that both ROS and marker gene expression in *sxi2* respond like the wild type.

PALD positively regulates XA21 protein levels and interacts with XA21 *in vivo*

LRR receptor-like kinases often function with an assortment of interacting proteins that modulate the activity or accumulation of the receptor (Lu et al., 2011). As loss of *XA21*-mediated resistance in *sxi2* could be due to a direct effect on the receptor, we performed immunoblot analysis to assess whether the knockout of PALD affects *XA21* gene expression or protein levels. XA21 protein levels in the two *PALD* native expression complementation lines (*pPALD:PALD/sxi2* nos. 1-4-1 and 2-1-1) and the overexpression complementation line *HA-PALD/sxi2* were compared with wild-type *XA21* and *sxi2* genotypes. We found that XA21 protein is significantly reduced in *sxi2* relative to wild-type *XA21* plants, and that the XA21 protein level is restored in all *PALD* complementation lines (Figure 4). These data suggest that PALD positively affects XA21 protein abundance. This effect is not due to altered transcript levels of the *XA21* gene, as transcript levels remain similar between complemented and mutant lines (Supplemental Figure 5).

To assess whether PALD associates with the XA21 protein *in vivo*, putative PALD interacting proteins were coprecipitated from leaf protein extracts of *Ubi:HA-PALD/sxi2* nos. 3-14-9 (hereafter referred to as *HA-PALD/sxi2)* using anti-HA beads. *XA21* plants, which do not carry any HA-tagged proteins, served as a negative control. Immunoblotting with anti-HA antibodies revealed two prominent bands near 140 kDa and one band around 100 kDa present in the input sample and enriched in the immunoprecipitation (IP) samples of *HA-PALD/sxi2* (Figure 5). These bands were not present in the *XA21* samples, indicating that the HA antibody is specific to HA-PALD. The two bands around 140 kDa are near the expected size of full-length PALD (142 kDa). Full-length Myc-XA21 protein ($\sim$140 kDa), detected by an anti-Myc antibody, is enriched in the IP samples of *HA-PALD/sxi2*. This result was further confirmed in an independent series of co-immunoprecipitation experiments in which XA21

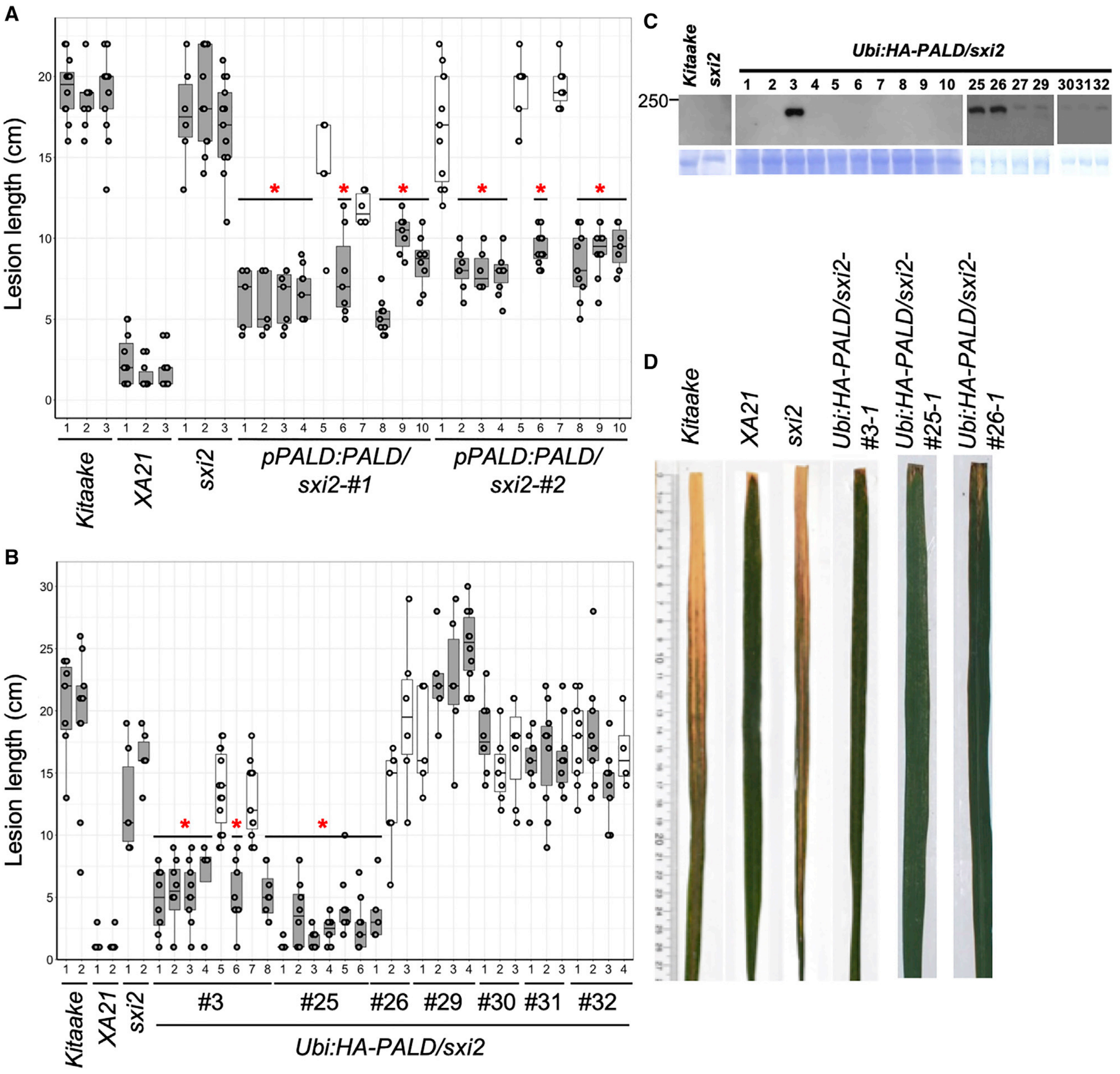

Figure 2. Ectopic expression of PALD protein rescues the susceptible phenotype of *sxi2*

(A) Genomic sequence of PALD complements the *sxi2* susceptible phenotype in T1 generation. Multiple leaves from individual wild-type, mutant, and complemented transgenic plants were inoculated with *Xoo* strain PXO99. Resulting lesion length data points were plotted on Whisker plots for *Kitaake*, *XA21*, *sxi2*, and *pPALD:PALD/sxi2* complementation lines 14 days post-inoculation (dpi; *n* = 10–16).

(B) Lesion lengths of the HA-PALD/sxi2 nos. 3, 25, 26, 29, 30, 31, and 32 T1 segregating plants. Three independent transgenic lines were inoculated by scissor clipping with *Xoo* strain PXO99 along with the control plants. Whisker plot represent the medium, upper, and lower quartiles of lesion measurements (cm) on *Kitaake*, *XA21*, *sxi2*, *Ubi:HA-PALD/sxi2* transgenic lines, *Ubi:HA-PALD/sxi2* null-segregants 14 dpi (*n* = 10–16). Asterisk indicates significant differences in lesion lengths of *Ubi:HA-PALD/sxi2* compared with parental *sxi2* plants.

(C) Photo of representative rice leaves shown in **(A)**.

(D) Immunoblot analysis of PALD protein in the T1 populations of 15 independent *Ubi:HA-PALD/sxi2* transgenic plants (line nos. 1, 2, 3, 4, 5, 6, 7, 8, 9, 10, 25, 26, 27, 29, 30, 31, and 32) using anti-HA antibody to detect PALD protein (145 kDa). After blotting, the membrane was stained with Coomassie brilliant blue as loading control (lower panel). Similar results were observed in one other independent experiment. *$P < 0.05$ compared with *sxi2* using Dunnett's test.

peptide fragments were detected in HA-PALD-enriched samples using liquid chromatography–tandem mass spectrometry (LC–MS/MS) (Supplemental Figure 6). These results demonstrate that HA-PALD interacts with XA21 *in vivo*.

The N terminus of PALD interacts with XA21 kinase and PALD C-terminal domain in yeast

To identify which PTP domain of PALD is essential for the interaction with XA21, we cloned several truncated *PALD* coding

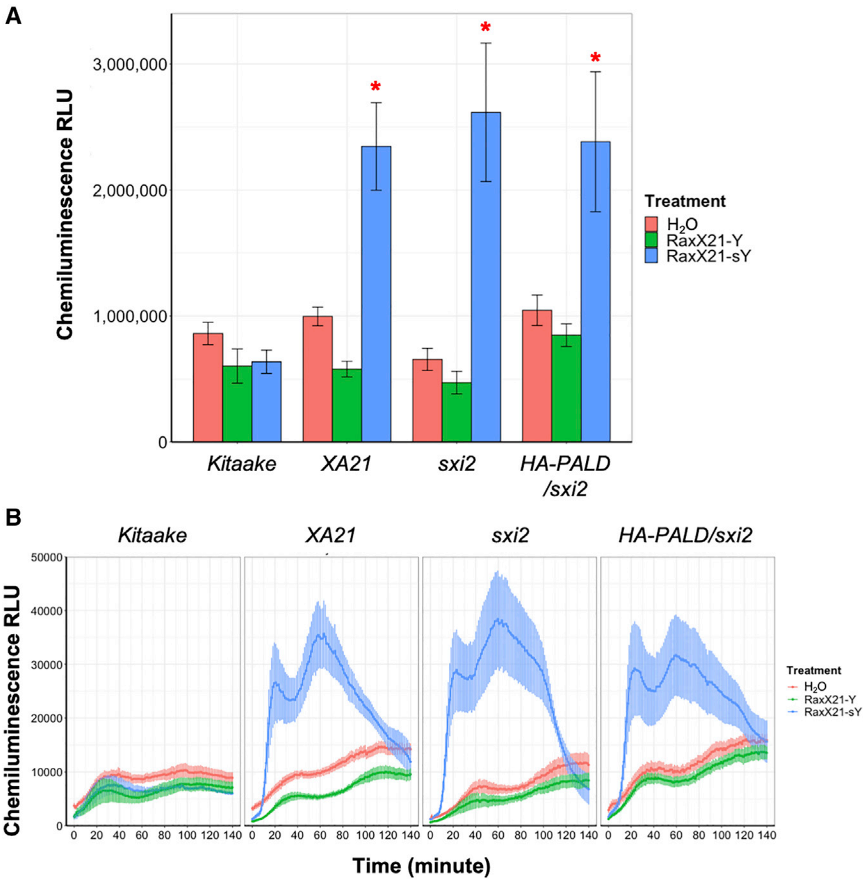

Figure 3. ROS burst is not compromised in *sxi2* plants

(A) ROS production in leaves of *Kitaake*, *XA21*, *sxi2*, and *HA-PALD/sxi2* treated with water, RaxX21-Y, or RaxX21-sY (500 nM). Data points depict mean ROS production ±SE (n = 6).

(B) Total chemiluminescence relative light units detected between 0 and 120 min of ROS assays shown in **(B)**. This experiment was performed three times with similar results. $^*P < 0.05$ compared with H_2O samples in each genotype using t-test.

sequences and the XA21 cytoplasmic kinase domain (XA21K668) in yeast two-hybrid vectors (Chen et al., 2010a). We observed that full-length PALD does not interact with XA21K668 in yeast two-hybrid, whereas the N-terminal half (PALD-AB) interacts with XA21K668 (Figure 6A and 6B). To determine if PTP-A or PTP-B is required for the interaction between PALD-AB and XA21K668, we further truncated PALD-AB from the C terminus (Figure 6C; from Del-1 to Del-5, A2, and A1) and N terminus (Figure 6D; from Del-6 to Del-10, and B). PALD-Del-3 and PALD-Del-4 show robust interactions with XA21K668, indicating that PTP-B domain is not required for the interaction. In contrast, all PALD N-terminal truncations affecting the PTP-A domain abolished the interaction between PALD and XA21K688, suggesting that the N terminus of PALD encompassing the PTP-A domain is required for interaction with XA21K668 (Figure 6D).

Plant PALD proteins contain three PTP domains (PTP-A, PTP-B, and PTP-C); in contrast, mammalian PALD proteins contain only two PTP domains (PTP-A and PTP-B). To study whether the PTP-C domain located at the C terminus may play a role in regulating plant PALD function, we first investigate if the PTP-C domain interacts PALD-AB and if it inhibits the interaction between PALD-AB and XA21K668. We tested whether the C-terminal end of the protein (from amino acid 701 to 1256) containing PTP-C domain interacts with PTP-AB protein truncation. The results suggest that the PTP-C domain interacts with PALD-AB; the interaction remains when PTP-B domain is gradually truncated from the C terminus (Figure 6E). In contrast, PALD-AB N-terminal truncations (Figure 6F) abolished interaction with PTP-C. Immunoblot analysis was performed to verify that all PALD truncations are expressed in the yeast two-hybrid assay (Supplemental Figure 7). The results show that full-length PALD is expressed at a lower level, while all the other PALD truncation forms are expressed at similar, higher levels. Together, these data show that the N terminus of PALD encompassing PTP-A is critical for interaction with the XA21 kinase domain and that the PTP-C domain interacts with the same PALD N terminus in the yeast two-hybrid.

XA21 kinase does not phosphorylate PALD *in vitro*

Structural-based homology modeling suggests that PALD possesses an iterative series of three subunits connected by linker regions (Supplemental Figure 8), each with one PTP domain. LC–MS/MS analysis of immunoprecipitated HA-PALD also identified three amino acid residues of the protein that can be phosphorylated *in vivo* (Supplemental Figure 8). Each PTP domain possesses one such residue, and Ser641 lies just outside the PTP-B predicted catalytic domain. Phosphorylated residues were identified in both mock- and RaxX21-sY-treated samples. Previously, we observed bidirectional phosphorylation between the XA21 kinase domain and OsSERK2, which is a positive regulator of XA21-mediated immunity that interacts with XA21 *in vivo* (Chen et al., 2014). Because PALD likely interacts with the XA21 intracellular kinase domain *in vivo* and can be phosphorylated, we hypothesized that XA21 might phosphorylate PALD. We assessed XA21 *in vitro* kinase activity using purified His-tagged XA21 kinase domain and full-length PALD recombinant proteins and radiolabeled [^{32}P]γ-ATP. Whereas the XA21 kinase autophosphorylates under the assay conditions, we found no evidence that the XA21 kinase transphosphorylates PALD when the proteins are incubated together (Supplemental Figure 9).

An active site mutation of PALD PTP-A abolishes complementation of resistance, while PTP-B is dispensable

Experimental analysis of PALD homologs in animal systems do not reach consensus whether PALD is a functional enzyme. While plant homologs possess three PTP domains (Supplemental Figure 10), only PTP-A and PTP-B possess the conserved cysteine residue in

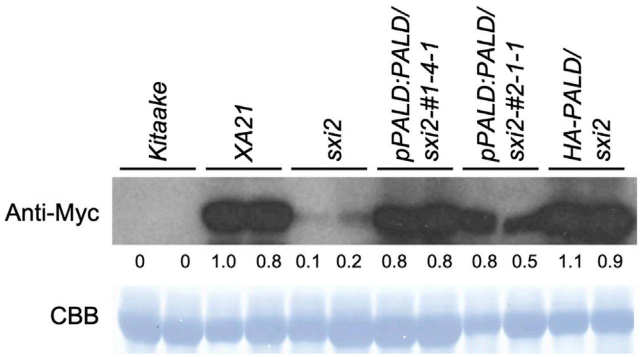

Figure 4. XA21 protein levels are reduced in the *sxi2* mutant

Myc-XA21 detection in total leaf protein extracts of independently transformed PALD complementation lines (*pPALD:PALD/sxi2* nos. 1 and 2) and one PALD overexpression line (*HA-PALD/sxi2*). Each genotype carries the *XA21* gene except for *Kitaake*, which is included as a negative control. Western blotting analysis with the anti-Myc antibody was used to detect Myc-XA21 protein levels. The XA21 protein level was quantitated and normalized to the Rubisco protein level; the value of the first XA21 sample was set as 1. The membrane was stained with Coomassie brilliant blue (CBB) for equal loading of the protein samples. The western blots were repeated three times with similar results.

the CXGXGRT active site motif typical of a phosphatase; the catalytic cysteine is of particular importance for initiating the enzymatic reaction (Bellomo et al., 2018). Our efforts to determine the phosphatase activity of PALD recombinant protein *in vitro* using the standard method of *para*-nitrophenyl phosphate as a substrate were unsuccessful (data not shown). To assess whether the putative phosphatase function of PALD is required *in planta* for resistance to *Xoo* strain PXO99, we introduced into *sxi2* a complementation construct containing native promoter-driven HA-tagged PALD either with the wild type, or with a cysteine-to-serine point mutation in PTP-A (C228S, abbreviated as CS1) or PTP-B (C647S, shown as CS2). Pathogen challenge of numerous independent T0 transgenic lines show that those with PALD mutated in PTP-A are unable to confer resistance to PXO99, while those with mutated PTP-B show restoration to *XA21* disease resistance similar to wild-type PALD (Figure 7). T1 segregation analyses confirmed the T0 results; null-segregant plants exhibit lesion lengths similar to *sxi2.* HA-PALD protein levels were assessed in the T0 generation, and detectable protein was present in these parent lines, in particular the PTP-A lines, indicating that loss of the ability to complement *sxi2* by the PTP-A protein is not due to the absence of the mutant PTP-A PALD protein. This result suggests that PALD possesses phosphatase activity *in vivo*, and that disruption of that activity by mutation of PTP-A abolishes the ability of PALD to complement *sxi2.* PTP-B appears dispensable for *XA21*-mediated immunity.

DISCUSSION

In this study, we report the identification and characterization of a fast-neutron-induced rice mutant, called *sxi2* (*suppressor of XA21-mediated immunity-2*). Through whole-genome resequencing, co-segregation analysis, and genetic complementation we show that a deletion of the *PALD* gene is responsible for the susceptible phenotype observed in the *sxi2* mutant. Introduction of PALD rescues the susceptible phenotype of *sxi2.*

What is the underlying cause of the *sxi2* susceptibility phenotype?

XA21 protein accumulation is reduced in the *sxi2* mutant. XA21 accumulation is restored in two PALD native expression complementation lines (*pPALD:PALD/sxi2* nos. 1 and 2) and three *PALD* overexpression lines that express detectable levels of PALD protein (*HA-PALD/sxi2* nos. 3, 25, and 26) (Figure 2C). Because *XA21* transcripts levels are similar in *XA21*, *sxi2*, and *HA-PALD/sxi2* plants (Supplemental Figure 5), we hypothesize that PALD affects XA21 protein accumulation but not transcript levels.

Although the XA21 protein level is reduced in *sxi2*, RaxX21-sY still induces many hallmarks of the immune response in *sxi2*, such as rapid ROS production and induction of defense marker genes (Figure 3 and Supplemental Figure 4). Our results suggest that, although the amount of XA21 protein in *sxi2* is sufficient to recognize RaxX21-sY, these responses are insufficient to confer resistance to *Xoo* strain PXO99. Together these data do not give a clear conclusion as to how mutations in PALD affect XA21-mediated signal transduction or XA21 protein accumulation.

How does PALD regulate XA21 immunity?

In vertebrates, PALD was reported to regulate chicken neural crest cell formation and migration (Adams et al., 2008; Gammill and Bronner-Fraser, 2002; Roffers-Agarwal et al., 2012). Because mutation of both catalytic cysteine residues within the predicted phosphatase active site of PALD did not abolish the function of PALD, the authors hypothesized that PALD may function as an anti-phosphatase that protects phosphosites from dephosphorylation (Roffers-Agarwal et al., 2012). In contrast, the expression levels of human PALD in an African green monkey kidney fibroblast-like cell line negatively correlated with insulin-stimulated AKT phosphorylation, suggesting that human PALD possesses bona fide phosphatase activity (Huang et al., 2009). Here, we report that the catalytic cysteine C228 in the PTP-A domain is required to restore resistance in *sxi2* (Figure 7), whereas C647 in the PTP-B domain is not essential. These results suggest that, in plants, the putative phosphatase activity of PALD is critical for function.

PALD possesses two conserved putative PTP domains, and interacts with XA21 *in vivo* in both rice and yeast (Figures 6 and 7). One possible explanation for the lower abundance of XA21 in *sxi2* is that PALD alters XA21 phosphorylation status directly. It has been shown that XA21 autophosphorylates Ser686, Thr688, and Ser689 residues localized in the juxtamembrane domain, and that alanine substitution of these residues specifically destabilizes the XA21 protein *in vitro* and *in vivo* (Xu et al., 2006). Transgenic rice carrying the same alanine substitutions in XA21 are slightly more susceptible to *Xoo* Philippine race 6 (PXO99), suggesting that autophosphorylation of these serine/threonine residues of XA21 positively regulate XA21 stability and immunity (Xu et al., 2006). In contrast, XA21 is capable of autophosphorylation of Tyr698, Tyr786, Tyr907, and Tyr909. Rice plants expressing phosphomimetic XA21 variants with these tyrosine residues mutated to aspartate ($XA21^{YD}$-GFP) are susceptible to *Xoo*. These phosphomimetic mutations do not affect XA21 protein levels, suggesting that these mutations directly compromise XA21 function. Conversely, rice plants expressing XA21-GFP fusion proteins with these tyrosine residues individually mutated to

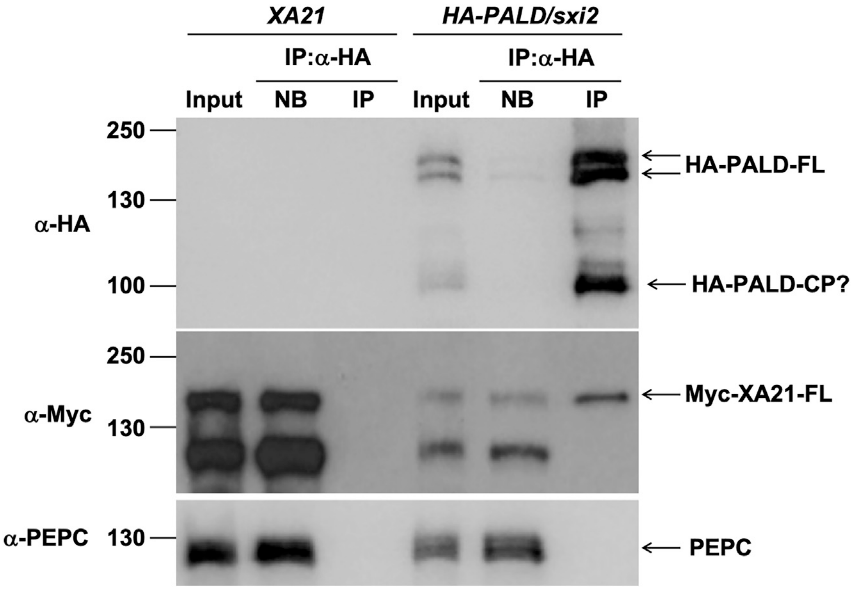

Figure 5. PALD and XA21 interact *in vivo*
Protein samples were extracted from leaves of XA21 and HA-PALD/sxi2. Myc-XA21 protein is expressed in both plants, while HA-PALD is only expressed in *HA-PALD/sxi2*. The protein crude extract was incubated with anti-HA beads and centrifuged to co-immunoprecipitate PALD. Proteins in the input, non-bound (NB) and immunoprecipitated (IP) samples were analyzed with SDS–PAGE and western blotting. One major band enriched in the immunoprecipitated samples around 100 kDa (HA-PALD-CP?) is a putative cleavage products of HA-PALD. Immunodetection of PEP carboxylase (PEPC) serves as a loading control. Note: the anti-Myc antibody detects a non-specific band around 120 kDa as reported previously (Park et al., 2008). Similar results were observed in two other independent experiments.

phenylalanine (XA21^{YF}-GFP), which prevents phosphorylation at these sites, maintain resistance to *Xoo* (Caddell et al., 2018). These results suggest that constitutive phosphorylation on these tyrosine residues of XA21 may negatively regulate *Xoo* resistance. Alternately, the effect of PALD may be indirect, influencing *XA21* immunity through interactions with not yet described components.

To distinguish between the direct versus indirect models, we carried out *in vitro* kinase assays to assess whether PALD is a direct phosphorylation target of XA21 or if the putative PALD phosphatase reduces XA21 phosphorylation. Although we did not observe PALD phosphorylation or XA21 dephosphorylation in these experiments (Supplemental Figure 9), this negative result is not conclusive as *in vitro* assays can give false negatives when essential components are missing.

PALD is widely conserved in plants and animals

Despite numerous studies of protein tyrosine kinases (PTKs) and PTPs in animal models, the biological functions of PTKs and PTPs in plants have only recently begun to be explored (Shankar et al., 2015). Human PTPs can be divided into three different families based on their critical catalytic amino acids: cysteine, aspartate, and histidine. The cysteine-based PTPs can be further categorized into three different classes, PTP family (class I), low-molecular-weight PTPs (class II), and cell division cycle 25s (class III) (Alonso and Pulido, 2016). Human *PALADIN* belongs to the class I PTP family with two PTP domains, and both domains carry the PTP signature motif CxxGxGR at their C terminus. PALD is the only gene in subclass IV of the class I PTP family (Alonso and Pulido, 2016). PALD is present as a single copy gene in rice and *Arabidopsis*, and homologs are found from vertebrates to plants (Supplemental Figure 10). PALD has not been previously studied in plants, where single to multiple homologs have been observed.

Using the structural homology predictive tool RaptorX, we generated a potential model for the three-dimensional PALD protein (Supplemental Figure 8). This analysis indicates that PALD is composed of three repeating subunits that each contains a PTP active site. Both PTP-A and PTP-B possess the proper corresponding amino acid residues on both the phosphate binding loop and acid loop found in common PTP enzymes. Despite this observed conservation, our genetic complementation of cysteine mutants in *sxi2* finds that only the cysteine of PTP-A is necessary for complementation (Figure 7). It may be that the PTP-A and PTP-B domains in rice PALD have a distinct function. Support for this hypothesis comes from a study of the two PTP domains of mammalian bi-domain PTP Leukocyte Common Antigen-Related (LAR). In this case, the conserved cysteine in the PTP-D1 domain of LAR is important for phosphatase activity, whereas the cysteine in the PTP-D2 domain of LAR functions in substrate specificity (Ahuja and Gopal, 2014; Tsujikawa et al., 2001).

PALD homologs in land plants carry a third PTP-like (PTP-C) subunit near the C terminus that is lacking in animal isoforms; PTP-C carries a serine instead of the typical cysteine residues in PTP-A and PTP-B, suggesting that the PTP-C domain does not have a tyrosine phosphatase activity. Instead, the PTP-C domain may inhibit the interaction between PALD-AB and XA21 by sequestering the PALD-AB domain (Figure 6). In support of this hypothesis, we observed three major bands of PALD in the immunoblots of *HA-PALD/sxi2*. The first two bands of 140 kDa are close to the predicted size of full-length PALD. The top band may be phosphorylated PALD, as we observed three phosphorylated residues on PALD *in vivo* (Supplemental Figure 6). The third band of 100 kDa might be a cleaved product of PALD carrying the first two PTP domains (Figure 5). This observation is reminiscent of the cleavage of human PTPα at the linker sequence between its two PTP domains (Sonnenburg et al., 2003). The linker region between the two PTP domains of PTPα is highly flexible and proteolytically labile, which may be important for the intra- and/or intermolecular interactions that are critical for its function. Our results showing that the N terminus of PALD is critical for the interactions with both XA21K668 and the C-terminal end of PALD and that deletion of PTP-C domain enhances the interaction between PALD and XA21 suggest that the

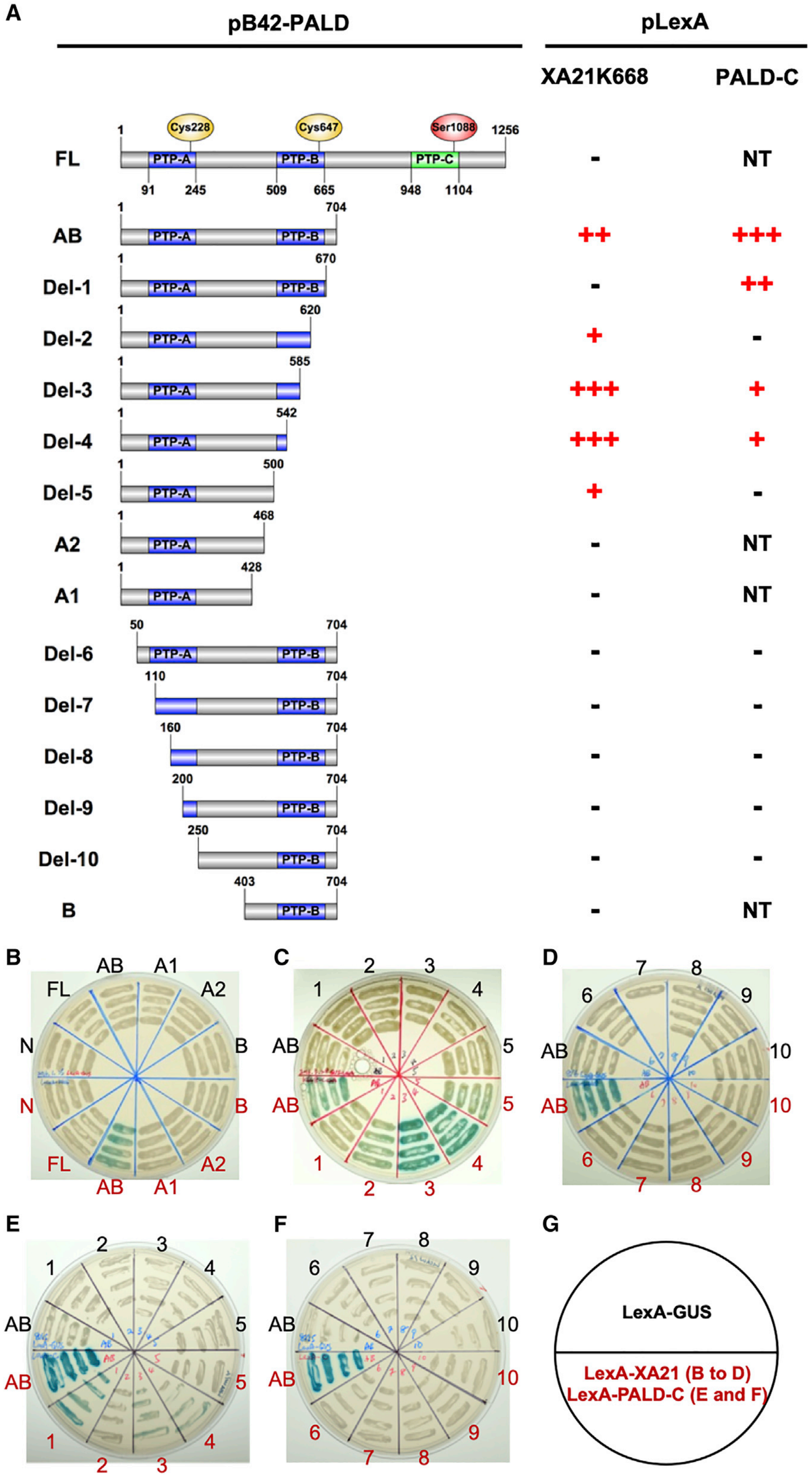

Figure 6. The PALD N-terminal domain interacts with both the XA21 kinase domain and the PALD C-terminal domain in yeast

(A) Summary of yeast two-hybrid results shown in **(B–F)**. Schematic structures of the PALD proteins are shown in the left panel. Full-length PALD (PALD-FL) and 14 different truncation forms of PALD were cloned into pB42 vector as indicated. The XA21 intracellular domain (XA21K668) and C-terminal end of PALD (PALD-C, carry 701-1256AA) were cloned in pLexA vector. β-Glucuronidase was cloned into the pLexA vector as negative control (pLexA-GUS). Yeast two-hybrid assays were performed to test the interaction between truncated PALD and XA21K668 **(B–D)**, and between truncated PALD and PALD-C **(E and F)**. The results of yeast two-hybrid are scored by the degree of blue color on colonies (–, no interaction; +, weak interaction; ++, moderate interaction; +++, strong interaction; NT, not tested).

(B–D) Pictures of yeast two-hybrid results showing the interactions between different PALD-AB truncation forms and XA21K668.

(E and F) Pictures of yeast two-hybrid results showing the interactions between different PALD-AB truncation forms and PALD-C.

(G) Plate design of the yeast two-hybrid assays shown in **(B–F)**.

PTP-C domain may inhibit the interaction between PALD-AB and XA21 by sequestering the PALD-AB domain (Figure 6).

In vertebrates, PALD has been reported to regulate insulin signaling (Huang et al., 2009), neural crest formation, and the apoptotic response (Egana et al., 2017). In a large-scale proteomic study, human PALD was found to interact with Toll-like receptor 9 (TLR9), DNA-dependent activator of IFN-regulatory factors (DAI), and interferon regulatory factor 7 (IRF7), all important regulators functioning in the human innate immune response (Li et al., 2011). The TLR9 receptor triggers the pro-inflammatory cytokine response upon the binding of DNA from bacteria and viruses. DAI and IRF7 are transcription factors involved in DNA-mediated activation of innate immune responses (Ning et al., 2011; Takaoka et al., 2007). Our finding that PALD plays a role in the XA21-mediated immune response provides a possible, but as yet undemonstrated, linkage between plant and animal defense pathways.

MATERIALS AND METHODS

Plant growth conditions and bacterial inoculation

Oryza sativa ssp. *Japonica* rice varieties Kitaake, a transgenic line of Kitaake carrying Myc-XA21 driven by maize ubiquitin promoter (*Ubi:Myc-XA21-Kitaake* line 7A-8, called *XA21* plants in this paper) (Park et al., 2010a), a fast-neutron mutant derived from XA21 (*suppressor of XA21-mediated immunity-2*, called *sxi2* in this paper), 2 PALD complementation lines in *sxi2* genetic background (*pPALD:PALD/sxi2*), 7 *RPB6* complementation lines in *sxi2* background (*pRPB6::RPB6/sxi2*), and 35 derived

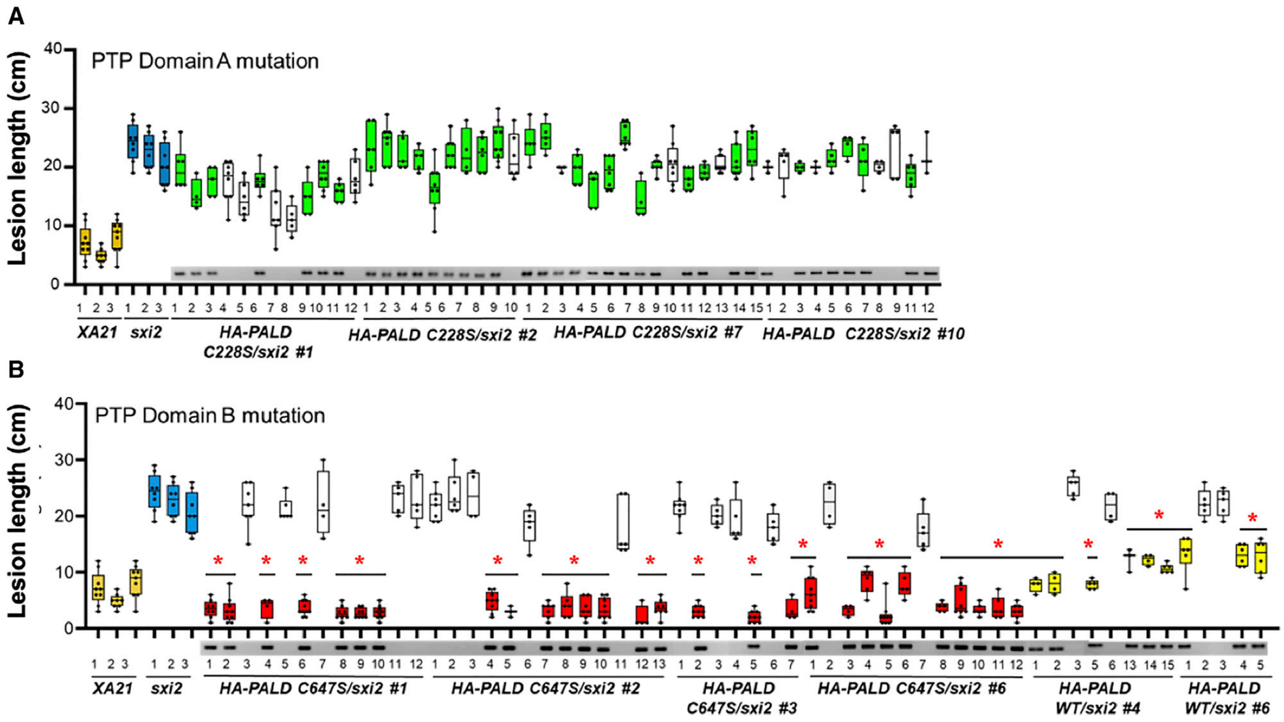

Figure 7. Mutation of the critical cysteine residue in PTP-A, but not PTP-B, of PALD abolishes complementation of disease resistance in *sxi2*

Multiple leaves from independent T1 transformed sxi2 plants transformed with PALD native promoter expression constructs driving expression of wild-type, PTP-A(C228S), or PTP-B(C647S) active site mutants were assessed for disease phenotype. Data represent the formation of disease lesions 14 days after inoculation with *Xoo* strain PXO99.

(A) Mutation of PALD PTP-A C228S abolished the complementation of *sxi2* resistance.

(B) Mutation of PALD PTP-B C647S retained the ability to complement the *sxi2* mutant, showing short lesions. Presence or absence of the transgene was detected by the presence of a PCR band. Null-segregant lines lacking the transgene are shown in white. **P* < 0.05 compared with *sxi2* using Dunnett's test.

transgenic line of *sxi2* carrying HA-PALD driven by ubiquitin promoter (*Ubi:HA-PALD/sxi2)* were used in these experiments. The transgenic lines *pPALD:PALD/sxi2* and *pRPB6::RPB6/sxi2* were generated using a mannose selection marker, while *Ubi:HA-PALD/sxi2* were generated using G418 selection. Kitaake plants do not contain *XA21* and display susceptible phenotypes to the *Xoo* strain PXO99 (Peng et al., 2008). Rice plants were grown in fertilizer water in a greenhouse for 5 weeks before inoculation (Pruitt et al., 2015; Wei et al., 2016). Five-week-old rice plants were inoculated with *Xoo* strain PXO99 using the scissor clipping method as described in previous studies (Song et al., 1995; Wei et al., 2016).

Assessment of gene expression

Detached rice leaves were treated with RaxX21-sY following a previous publication with minor modifications (Pruitt et al., 2015). In brief, rice seeds of Kitaake, XA21, *sxi2*, and HA-PALD/sxi2 were first germinated in water and grown in hydroponic system using A-OK Starter Plugs (Grodan) and watered with Hoagland's solution (pH 5.7) once a week. Chemically synthesized RaxX21-sY (aa 35–55, called RaxX-sY in this study) is used as described in a previous publication (Pruitt et al., 2015). For the gene expression analysis, rice leaf strips were cut into 2-cm-long strips and incubated in distilled water overnight to reduce wounding responses. The incubation water was then replaced with 500 nM RaxX21-sY peptide or water (mock treatment) to start peptide treatment. Leaf samples were then collected and snap frozen in liquid N_2 1 and 8 h after the treatment for gene expression assays. For gene expression assays, total RNAs were extracted following lab protocol (Pruitt et al., 2015).

ROS assay

ROS assays were performed as described previously with minor differences (Pruitt et al., 2015; Wei et al., 2016). Leaves of 4–5-week-old hydroponically grown rice plants were cut longitudinally along the mid vein and then transversely cut into 1- to 1.5-mm-thick leaf pieces. These leaf pieces were incubated on autoclaved ddH_2O in regular Petri dishes overnight with constitutive light (between 5 and 10 μmol/(m2*s)) at room temperature. The next morning, two leaf pieces were transferred into each well of a 96-well white plate containing 95 μl of excitation solution (5–20 mM L-012 [Wako] and horseradish peroxidase [0.5–2 mg/ml; Sigma]). Five μL of 10 mM sulfated (RaxX21-sY), non-sulfated (RaxX21-Y) peptides, or ddH_2O were added into each well right before the start of the ROS assay. Chemiluminescence was measured for 3 h with 0.5 s per reading with a high-sensitivity plate reader (TriStar, Berthold).

RNA extraction and qRT–PCR analysis

Rice leaves were homogenized to powder using a tissue lyser and RNA were extracted with 1 ml of TRIzol Reagent (Thermo Fisher) following the manufacturer's protocol. RNA samples were treated with Turbo DNase (Ambion) at 37°C for 60 min to remove genomic DNA contamination. RNA samples were purified using a NucleoSpin RNA column (Macherey-Nagel) and stored in −20°C refrigerator. For each RNA sample, 2 μg of total RNA were used for cDNA synthesis using the High Capacity cDNA Reverse Transcription Kit (Thermo Fisher). Gene expression changes were determined using the ΔΔCt method and normalized to actin

(LOC_Os03g50885) and compared with mock-treated samples (Schmittgen and Livak, 2008).

Protein extraction and western blotting

Total proteins extracted from rice leaves were analyzed by western blotting as described previously (Wei et al., 2016). In brief, around 70 mg of leaf tissues were collected in a 2-ml homogenization tube and snap frozen in liquid N_2. Leaf samples were homogenized into powder and incubated with 200 μl of protein extraction buffer on ice for 30 min (150 mM NaCl, 10 mM sodium phosphate buffer [pH 7.2], 2 mM EDTA, 1% Triton X-100, 1 mM PMSF, 0.07% β-mercaptoethanol, Protease Inhibitor Cocktail P-9599 [Sigma], and 20 mM NaF). Cell debris was removed by centrifugation with 16 100 *g*, 20 min at 4°C twice. Protein concentrations were measured using the Bradford method and normalized to the lowest sample concentrations in each experiment. Equal amounts of total proteins were mixed with 5× protein sample buffer and incubated at 95°C for 10 min before SDS–PAGE. For each well, around 40 μg of proteins were loaded and separated by SDS–PAGE. Proteins were transferred from the gel to polyvinylidene difluoride (PVDF) membrane for western blotting analysis. Three different antibodies were used in this study: anti-HA (1:3000 dilution, Covance catalog no. MMS-101R); anti-Myc (1:1000 dilution, Santa Cruz sc-47694); and anti-PEPC (1:2000 dilution, Abcam catalog no. ab34793)

Co-immunoprecipitation assay

Total proteins were extracted from rice leaves of 5-week-old XA21 and HA-PALD/sxi2 plants in the absence of *Xoo* or RaxX21-sY. Leaves of XA21 and HA-PALD/sxi2 plants were collected in liquid N_2 and kept at –80°C until ready. Around 500 mg of leaves were grounded in N_2 by mortar and pestle. Leaf powders were incubated with 1.5 ml of protein extraction buffer on ice for 30 min (150 mM NaCl, 10 mM sodium phosphate buffer [pH 7.2], 2 mM EDTA, 1% Triton X-100, 1 mM PMSF, 0.07% β-mercaptoethanol, Protease Inhibitor Cocktail P-9599 [Sigma], and 20 mM NaF). Cell debris was removed by centrifugation with 16 100 *g*, 20 min at 4°C twice. PALD proteins were precipitated by adding 30 μl of Roche Anti-HA Affinity Matrix (Roche). The protein extracts were incubated with anti-HA affinity resin to retain HA-PALD and its associated proteins. Protein samples were separated via SDS–PAGE and probed by anti-HA and anti-Myc antibodies sequentially, which specifically targets HA-PALD and Myc-XA21 proteins, respectively. The protein samples were incubated at 4°C overnight on an end-to end rotator. The samples were centrifuged at 1500 *g* for 3 min at 4°C to precipitated HA-PALD and protein complex. The pellets were washed five times with cold protein extraction buffer twice to remove unbounded proteins. SDS–PAGE sample buffer was added to the pellet and incubated at 95°C for 10 min. After centrifugation at 16 100 *g* for 5 min, 20 μl of the supernatant were loaded into 8% acrylamide gel for SDS–PAGE. Proteins were transferred to PVDF membrane at 23 V overnight at 4°C. Myc-XA21 proteins were detected by western blotting analysis with c-Myc antibody (Santa Cruz, sc-47694, 1:1000 dilution) and goat anti-mouse IgG-HRP (Santa Cruz, sc-2005, 1:2000 dilution). The PVDF membrane was incubated with 1 ml of Pierce ECL 2 western blotting substrate (Thermo Scientific) and visualized with X-ray film.

Purification of recombinant proteins and *in vitro* kinase assay

Purification of His-Nus fusion proteins and *in vitro* kinase assays were performed as described previously (Schwessinger et al., 2011). In brief, the full length of PALD CDS was cloned in pDEST17 vector with a His-tag in the N terminus. The His-Nus-XA21JK plasmid was used in a previous study (Chen et al., 2014). His-Nus-XA21JK and His-PALD plasmids were transformed into BL21 cells for protein expressions. After being induced by isopropyl β-d-1-thiogalactopyranoside, proteins were purified from BL21 cells using Ni-NTA agarose beads following the manufacturer's protocol. For *in vitro* kinase assay, 3 μg of XA21K668 and PALD-AB proteins were incubated in 20 μl kinase buffer (50 mM Tris [pH 7.5], 10 mM $MgCl_2$, 10 mM $MnCl_2$, 1 mM DTT) in the presence of 1 μM unlabeled ATP or 10 μCi of [^{32}P]γ-ATP for 30 min at 30°C with shaking at 900 rpm. The reactions were stopped by adding 5× protein sample buffer and boiled for 5 min. The phosphorylation status of fusion proteins was analyzed by autoradiography after separation of the *in vitro* kinase assay by 8% SDS–PAGE.

Yeast two-hybrid assay

Yeast two-hybrid experiments were carried out using the Matchmaker LexA yeast two-hybrid system (Clontech) as described in a previous study (Chen et al., 2014). The cDNA of PALD was first cloned in pENTR vector and sequenced. The full-length PALD cDNA sequence was used as a template to clone the truncation forms of PALD construct. PALD-AB and PALD-C are subcloned constructs that carry the N-terminal half (PALD-AB, aa 1–704) and the C-terminal half (PALD-C, aa 701–1256) of PALD, respectively. The other 13 truncation forms of PALD were subcloned from PALD-AB. XA21K668 was cloned and used in a previous study (Chen et al., 2014). PALD and XA21K668 cDNA were then subcloned into pB42 (PALD-pB42) and pLexA (XA21K668-pLexA) vectors, respectively. Both of PALD-pB42 and XA21K668-pLexA vectors were co-transformed into yeast EGY48 strain (Clontech) by using the Frozen-EZ Yeast Transformation II Kit (Zymo Research) and spread on an appropriate auxotrophic medium. For negative controls, the pLexA-XA21K668 was replaced by pLexA-GUS plasmid.

SUPPLEMENTAL INFORMATION

Supplemental information can be found online at *Plant Communications Online*.

FUNDING

This work was supported by the following grants to P.R.: NIH no. GM59962, NIH no. GM122968, NSF no. 1237975, NSF IOS-1656501, and NSF-NIFA no. 2017-03128. This work was supported by the following grant to T.-C.C.: a Taiwan Government Scholarship. Support for M.S. was provided by the Corteva Open Innovations Program. The work conducted by the Joint BioEnergy Institute was supported by the Office of Science, Office of Biological and Environmental Research of the U.S. Department of Energy under contract no. DE-AC02-05CH11231.

AUTHOR CONTRIBUTIONS

T.-C.C., M.C., M.S., and P.R. conceived and designed the experiments, analyzed the data, and wrote the paper. T.-C.C., M.C., R.R., M.S., Y.W., and A.I. performed the experiments, analyzed the data, and/or prepared figures and tables. All authors reviewed drafts of the paper.

ACKNOWLEDGMENTS

No conflict of interest declared.

Received: March 18, 2021
Revised: June 16, 2021
Accepted: June 27, 2021
Published: June 29, 2021

REFERENCES

Adams, M.S., Gammill, L.S., and Bronner-Fraser, M. (2008). Discovery of transcription factors and other candidate regulators of neural crest development. Dev. Dyn. **237**:1021–1033.

Ahuja, L.G., and Gopal, B. (2014). Bi-domain protein tyrosine phosphatases reveal an evolutionary adaptation to optimize signal transduction. Antioxid. Redox Signal. **20**:2141–2159.

Alonso, A., and Pulido, R. (2016). The extended human PTPome: a growing tyrosine phosphatase family. FEBS J. **283**:1404–1429.

Bellomo, E., Abro, A., Hogstrand, C., Maret, W., and Domene, C. (2018). Role of zinc and magnesium ions in the modulation of phosphoryl transfer in protein tyrosine phosphatase 1B. J. Am. Chem. Soc. **140**:4446–4454.

Boutrot, F., and Zipfel, C. (2017). Function, discovery, and exploitation of plant pattern recognition receptors for broad-spectrum disease resistance. Annu. Rev. Phytopathol. **55**:257–286.

Caddell, D.F., Wei, T., Sharma, S., et al. (2018). Four tyrosine residues of the rice immune receptor XA21 are not required for interaction with the co-receptor OsSERK2 or resistance to *Xanthomonas oryzae* pv. *oryzae*. PeerJ **6**:e6074.

Chen, X., et al. (2010). A conserved threonine residue in the juxtamembrane domain of the XA21 pattern recognition receptor is critical for kinase autophosphorylation and XA21-mediated immunity. J. Biol. Chem. **285**:10454–10463.

Chen, X., Zuo, S., Schwessinger, B., et al. (2014). An XA21-associated kinase (OsSERK2) regulates immunity mediated by the XA21 and XA3 immune receptors. Mol. Plant **7**:874–892.

Couto, D., and Zipfel, C. (2016). Regulation of pattern recognition receptor signalling in plants. Nat. Rev. Immunol. **16**:537–552.

Egana, I., Kaito, H., Nitzsche, A., et al. (2017). Female mice lacking Pald1 exhibit endothelial cell apoptosis and emphysema. Sci. Rep. **7**:15453.

Gammill, L.S., and Bronner-Fraser, M. (2002). Genomic analysis of neural crest induction. Development **129**:5731–5741.

Huang, S.M.A., Hancock, M.K., Pitman, J.L., Orth, A.P., and Gekakis, N. (2009). Negative regulators of insulin signaling revealed in a genome-wide functional screen. PLoS One **4**:e6871.

Kim, Y.J., Wang, R., Gao, L., et al. (2016). POWERDRESS and HDA9 interact and promote histone H3 deacetylation at specific genomic sites in *Arabidopsis*. Proc. Natl. Acad. Sci. U S A **113**:14858–14863.

Li, S., Wang, L., Berman, M., Kong, Y.Y., Dorf, M.E., et al. (2011). Mapping a dynamic innate immunity protein interaction network regulating type I interferon production. Immunity **35**:426–440.

Li, G., Jain, R., Chern, M., et al. (2017). The sequences of 1504 mutants in the model rice variety Kitaake facilitate rapid functional genomic studies. Plant Cell **29**:1218–1231.

Lu, D., Lin, W., Gao, X., et al. (2011). Direct ubiquitination of pattern recognition receptor FLS2 attenuates plant innate immunity. Science **332**:1439–1442.

Luu, D.D., Joe, A., Chen, Y., et al. (2019). Biosynthesis and secretion of the microbial sulfated peptide RaxX and binding to the rice XA21 immune receptor. Proc. Natl. Acad. Sci. U S A **116**:8525–8534.

Macho, A.P., and Zipfel, C. (2014). Plant PRRs and the activation of innate immune signaling. Mol. Cell **54**:263–272.

Ning, S., Pagano, J.S., and Barber, G.N. (2011). IRF7: activation, regulation, modification and function. Genes Immun. **12**:399–414.

Park, C.J., Peng, Y., Chen, X., et al. (2008). Rice XB15, a protein phosphatase 2C, negatively regulates cell death and XA21-mediated innate immunity. Plos Biol. **6**:e231.

Park, C.J., et al. (2010). Ectopic expression of rice Xa21 overcomes developmentally controlled resistance to *Xanthomonas oryzae* pv. *oryzae*. Plant Sci. **179**:466–471.

Park, C.J., Caddell, D.F., and Ronald, P.C. (2012). Protein phosphorylation in plant immunity: insights into the regulation of pattern recognition receptor-mediated signaling. Front Plant Sci. **3**:177.

Peng, Y., Bartley, L.E., Chen, X., et al. (2008). OsWRKY62 is a negative regulator of basal and Xa21-mediated defense against *Xanthomonas oryzae* pv. *oryzae* in rice. Mol. Plant **1**:446–458.

Pruitt, R.N., Schwessinger, B., Joe, A., et al. (2015). The rice immune receptor XA21 recognizes a tyrosine-sulfated protein from a Gram-negative bacterium. Sci. Adv. **1**:e1500245.

Roffers-Agarwal, J., Hutt, K.J., and Gammill, L.S. (2012). Paladin is an antiphosphatase that regulates neural crest cell formation and migration. Dev. Biol. **371**:180–190.

Schmittgen, T.D., and Livak, K.J. (2008). Analyzing real-time PCR data by the comparative C(T) method. Nat. Protoc. **3**:1101–1108.

Schwessinger, B., Roux, M., Kadota, Y., et al. (2011). Phosphorylation-dependent differential regulation of plant growth, cell death, and innate immunity by the regulatory receptor-like kinase BAK1. PLoS Genet. **7**:e1002046.

Shankar, A., Agrawal, N., Sharma, M., Pandey, A., Pandey, G.K., et al. (2015). Role of protein tyrosine phosphatases in plants. Curr. Genomics **16**:224–236.

Song, W.Y., Wang, G.L., Chen, L.L., et al. (1995). A receptor kinase-like protein encoded by the rice disease resistance gene, Xa21. Science **270**:1804–1806.

Sonnenburg, E.D., Bilwes, A., Hunter, T., and Noel, J.P. (2003). The structure of the membrane distal phosphatase domain of RPTPalpha reveals interdomain flexibility and an SH2 domain interaction region. Biochemistry **42**:7904–7914.

Takaoka, A., Wang, Z., Choi, M.K., et al. (2007). DAI (DLM-1/ZBP1) is a cytosolic DNA sensor and an activator of innate immune response. Nature **448**:501–505.

Tsujikawa, K., Kawakami, N., Uchino, Y., et al. (2001). Distinct functions of the two protein tyrosine phosphatase domains of LAR (leukocyte common antigen-related) on tyrosine dephosphorylation of insulin receptor. Mol. Endocrinol. **15**:271–280.

Wei, T., Chen, T.C., Ho, Y.T., and Ronald, P.C. (2016). Mutation of the rice XA21 predicted nuclear localization sequence does not affect resistance to *Xanthomonas oryzae* pv. *oryzae*. PeerJ **4**:e2507.

Xu, W.H., Wang, Y.S., Liu, G.Z., et al. (2006). The autophosphorylated Ser686, Thr688, and Ser689 residues in the intracellular juxtamembrane domain of XA21 are implicated in stability control of rice receptor-like kinase. Plant J. **45**:740–751.

Plant Communications, Volume *2*

Supplemental information

Paladin, a tyrosine phosphatase-like protein, is required for *XA21*-mediated immunity in rice

Tsung-Chi Chen, Mawsheng Chern, Michael Steinwand, Deling Ruan, Yu Wang, Arkin Isharani, and Pamela Ronald

Paladin (_PALD_), a tyrosine phosphatase-like protein, is required for _XA21_-mediated immunity in rice

Tsung-Chi Chen*, Mawsheng Chern*, Michael Steinwand, Deling Ruan, Yu Wang, Arkin Isharani, and Pamela Ronald

Abstract

XA21 encodes a rice immune receptor that confers robust resistance to most strains of the Gram-negative bacterium *Xanthomonas oryzae* pv. *oryzae* (*Xoo*). *XA21*-mediated immunity is triggered by recognition of a small protein called RaxX-sY (required for activation of *XA21*-mediated immunity X, tyrosine-sulfated) secreted by *Xoo.* To identify components regulating *XA21*-mediated immunity, we generated and screened a mutant population of fast neutron mutagenized rice expressing *Ubi:Myc-XA21* for those susceptible to *Xoo*. Here, we report the characterization of one of these rice mutants, named *sxi2* (*suppressor of XA21-mediated immunity-2*). Whole genome sequencing reveals that *sxi2* carries a deletion of the *PALADIN* (*PALD*) gene that encodes a protein with three putative protein tyrosine phosphatase-like domains (PTP-A, B, and C). Expression of *PALD* in the *sxi2* genetic background is sufficient to complement the susceptible phenotype, and requires the catalytic cysteine of the PTP-A active site to restore resistance. PALD co-immunoprecipitates with full-length XA21. XA21 protein levels are positively regulated by the presence of the *PALD* transgene. We find that *sxi2* retains many hallmarks of XA21-mediated immunity similar to wild type. These results demonstrate a function for PALD, a previously uncharacterized class of phosphatase, in rice innate immunity, and the requirement of the conserved cysteine in the PTP-A domain for PALD function.

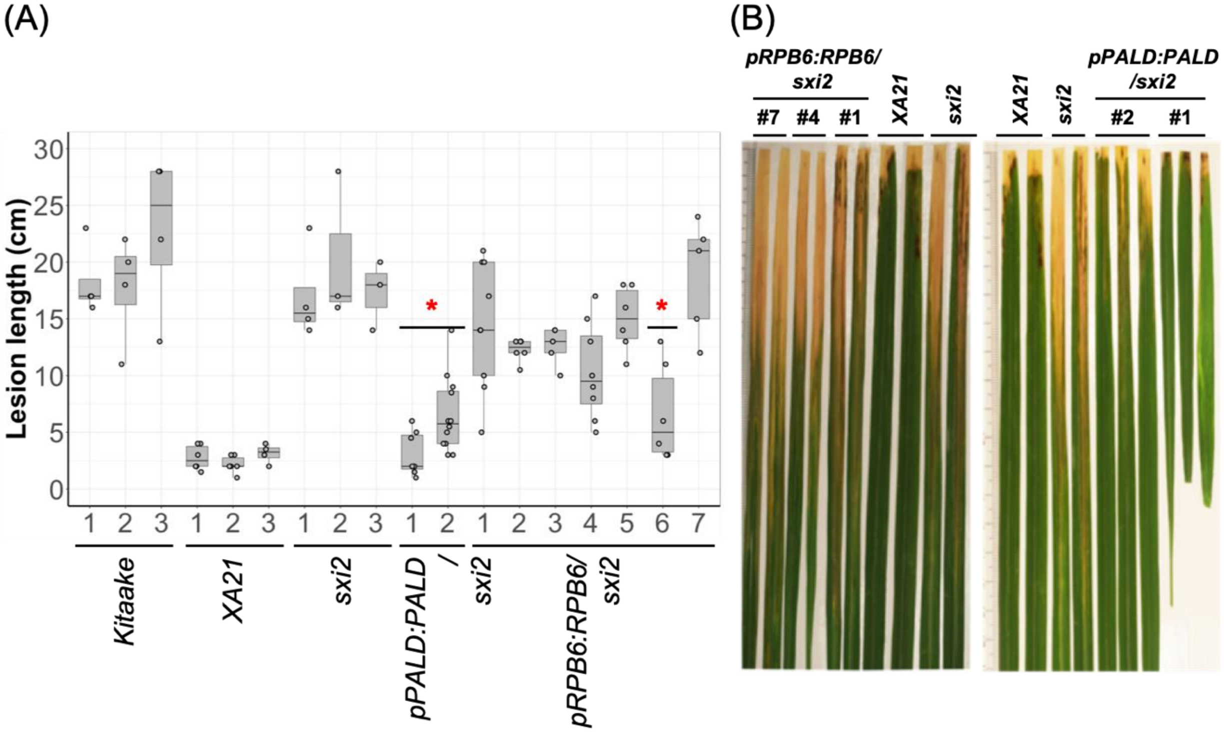

Supplemental Figure 1. The PALD gene complements the *sxi2* susceptible phenotype in T0 plants. **(A)** Lesion lengths of two *pPALD:PALD/sxi2* and seven *pRPB6:RPB6/sxi2* T0 complementation lines. Three independent transgenic lines were inoculated by scissor clipping with *Xoo* strain PXO99 along with the control plants. Whisker plot and represent the medium, upper and lower quartiles of lesion measurements (cm) on Kitaake, XA21, *sxi2*, *pPALD:PALD/sxi2* complementation lines, and *pRPB6::RPB6/sxi2* complementation lines 14 d post-inoculation. **(B)** Image of the inoculation results of **(A).** *P < 0.05 compared with *sxi2* using Dunnett's test.

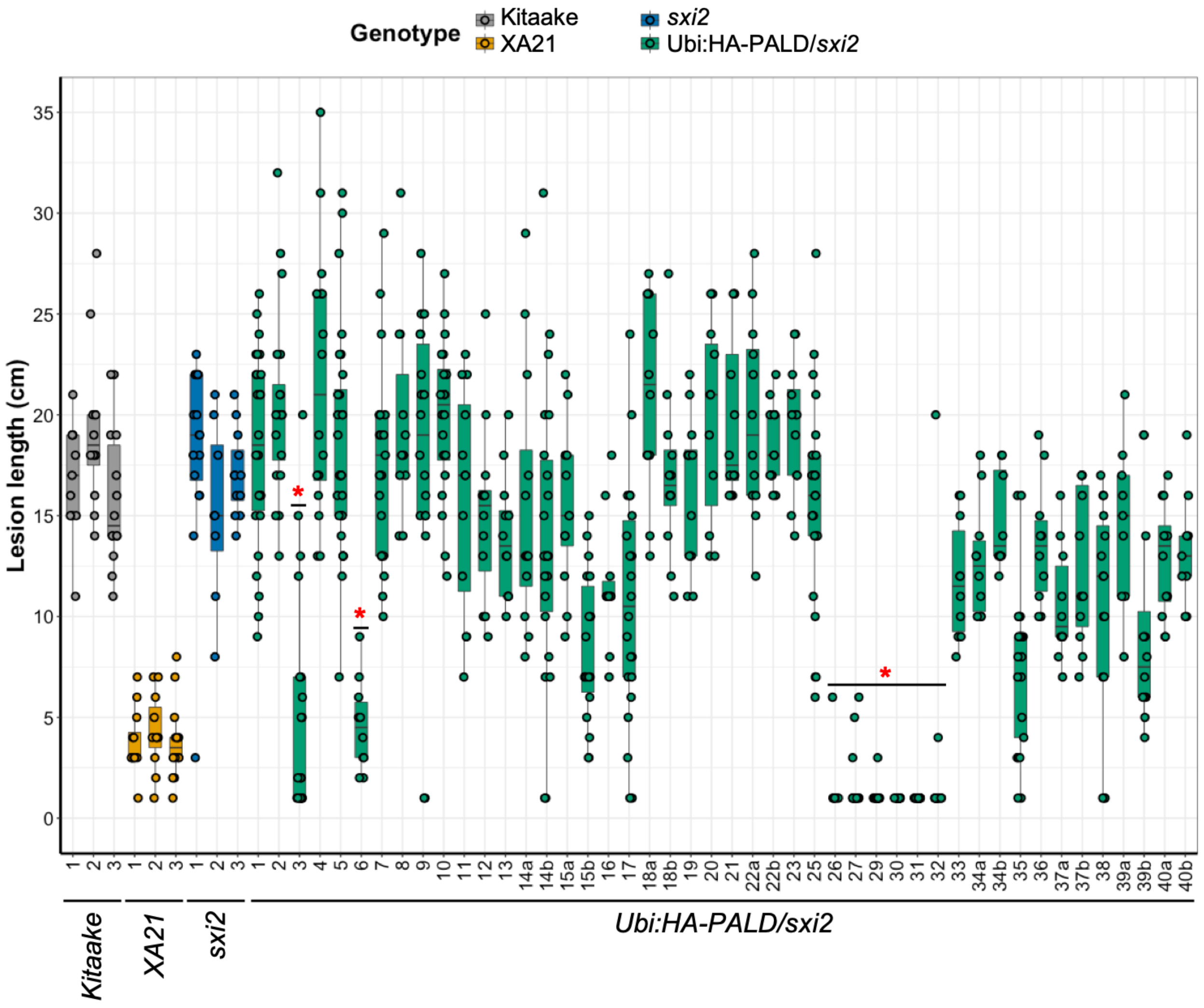

Supplemental Figure 2. Lesion lengths of *Ubi:HA-PALD/sxi2* T0 plants after *Xoo* inoculation. To test if the overexpression of PALD rescues the susceptible phenotypes of *sxi2*, 38 independent T0 plants (*Ubi:HA-PALD/sxi2*) were inoculated with *Xoo* strain PXO99. Lesion lengths were measured two weeks after the inoculation. Rice seeds from the tillers showing resistant phenotypes (with shorter than 8 cm of lesion length) were harvested and inoculated again in the T1 generation to test if the resistant phenotypes are heritable. The inoculation results of T1 plants derived from T0 lines #3, #25, #26, #29, #30, #31, and #32 are shown in Figure 2B. *P < 0.05 compared with *sxi2* using Dunnett's test.

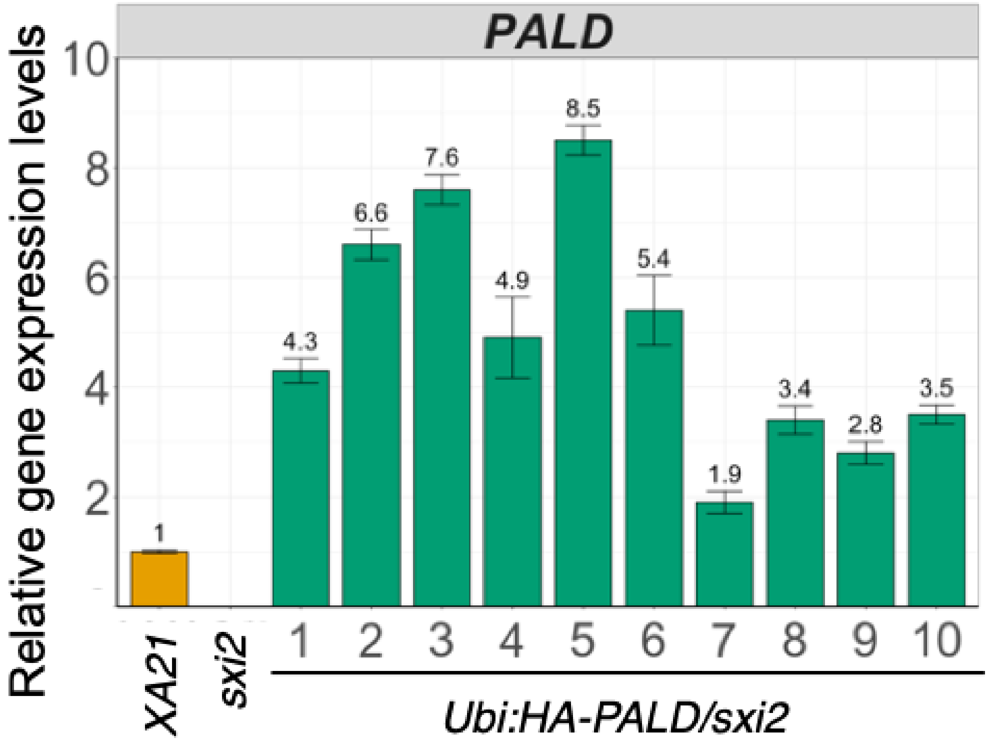

Supplemental Figure 3. Quantitative RT-PCR analysis of *PALD* expression levels in the leaves of 10 independent T0 populations of Ubi:HA-PALD/sxi2 transgenic plants. Quantitative RT-PCR analysis of *PALD* expression levels in the leaves of T0 populations of 10 independent Ubi:HA-PALD/sxi2 transgenic plants (from line #1 to #10). The expression level of *PALD* is normalized to the XA21 control. PALD expression levels in all Ubi:HA-PALD/sxi2 plants are significantly higher than XA21 ($P < 0.05$, Student's t-test). This experiment was done once with three biological replicates.

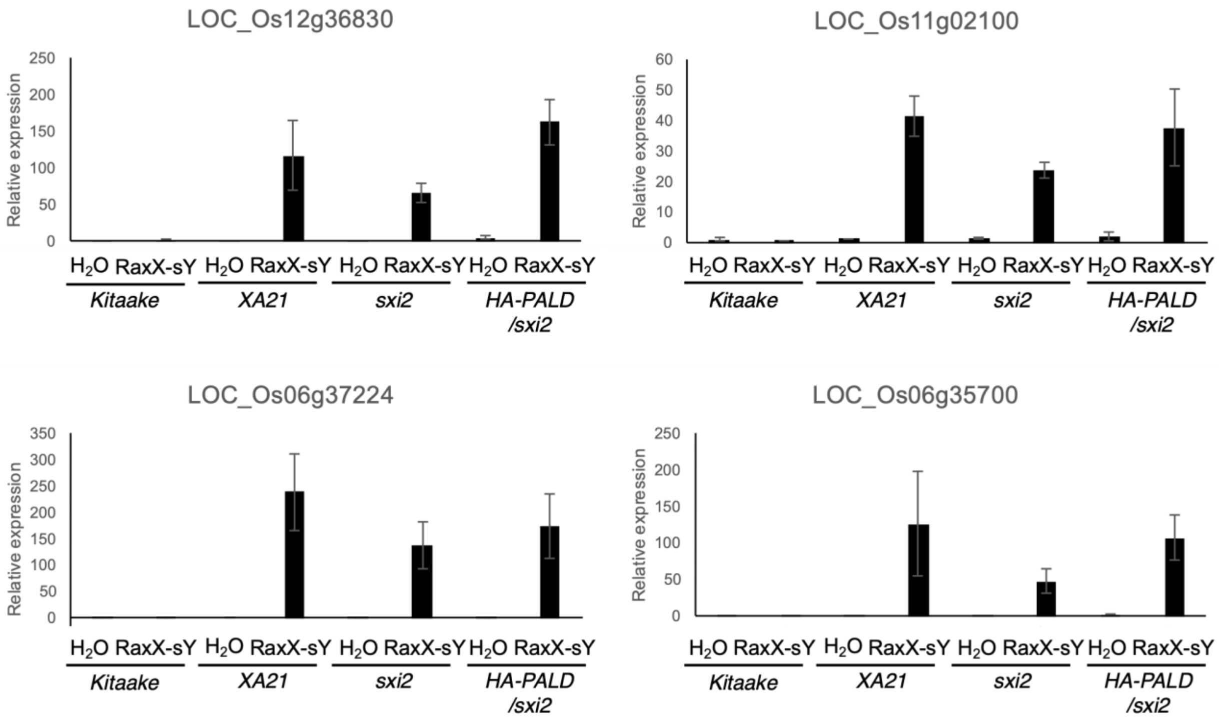

Supplemental Figure 4. Four defense marker genes are up-regulated in the three XA21-carrying genotypes. The relative expression levels of four defense marker genes after 8 hours of RaxX-sY or H_2O treatment. The expression levels of these genes are normalized to the Kitaake-H_2O samples. Bars and error bars represent the mean ± SD of relative expression levels in Kitaake, XA21, *sxi2*, *HA-PALD/sxi2* transgenic lines 8 hours after treatment (n = 3).

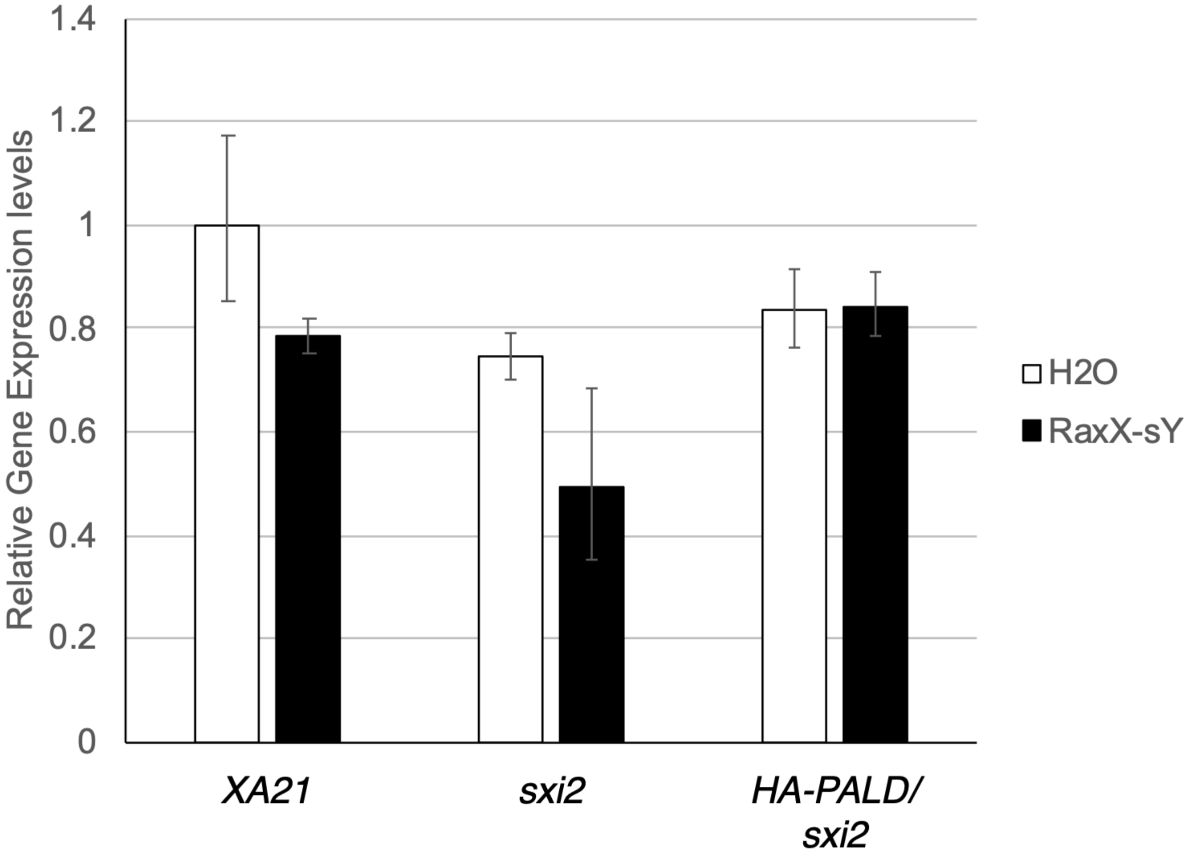

Supplemental Figure 5. qRT-PCR analysis of XA21 expression levels among the three XA21-carrying genotypes. Transcript levels of *XA21* in leaves of *XA21*, *sxi2*, and *HA-PALD/sxi2* rice plants treated with H_2O (mock) or RaxX21-sY (500 nM, n = 3) for 8 hours. All data points depict means ± SE. These experiments were repeated three times with similar results.

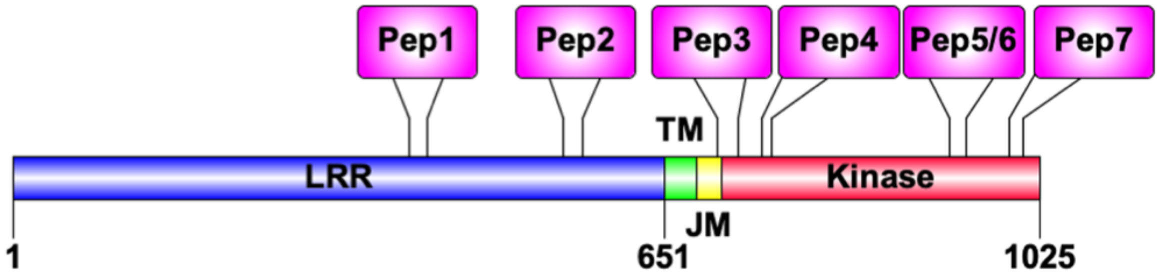

Peptide	Sequence	Number of Hits_rep1	Number of Hits_rep2	Number of Hits_rep3
1	DIGNLIGLQHLYLcNNNFR	1		
2	YLYLQNNLLSGSIPSALGQLK	1		
3	ATDGFAPTNLLGSGSFGSVYK	2		
4	SFTAEcEALR	1	1	
5	QYVELGLHGR		1	
6	VTDVVDTK	1		
7	TPTGDIIDELNAIK	1	2	1

Supplemental Figure 6. Liquid chromatography-mass spectrometry analysis identifies XA21 as an *in vivo* interactor of PALD. Co-immunoprecipitated HA-PALD samples were subjected to analysis with LC-MS/MS to identify putative interactors. Seven peptides of the XA21 receptor were identified, matched to the LRR and kinase protein domains. In three replicate samples from separate experiments, peptides matched to the kinase domain recur most often.

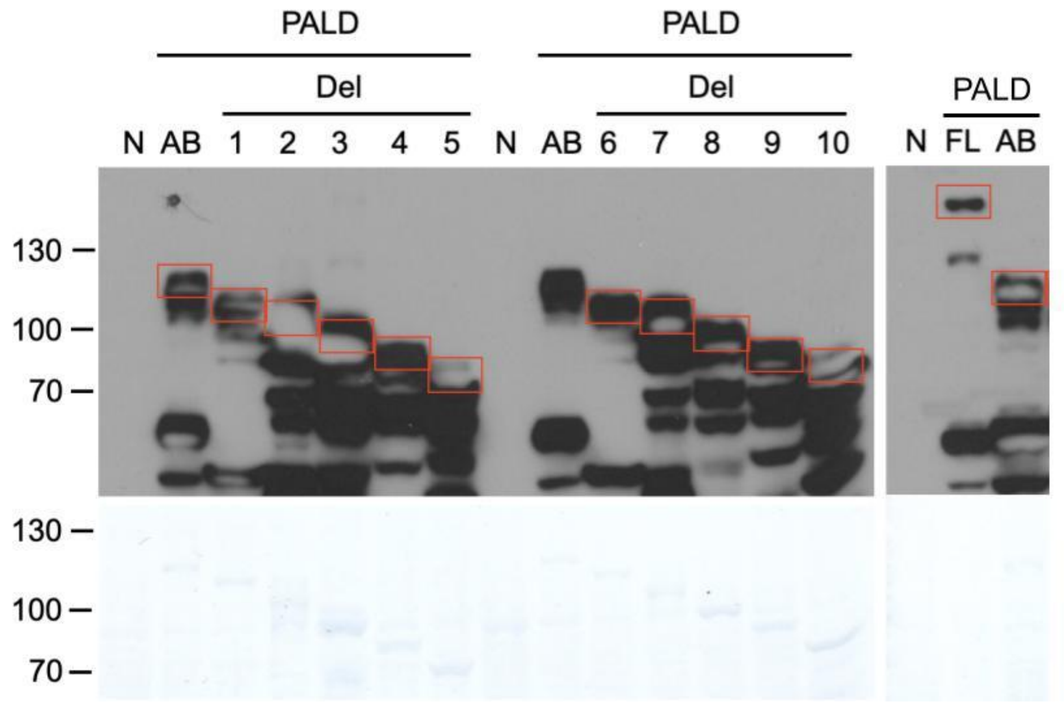

Supplemental Figure 7. Western blot analysis of PALD proteins expressed in yeast. Upper panel: We performed Western blot analysis to measure the expression of different truncation forms of PALD-HA proteins used in the Yeast two-hybrid assays (Figure 6). Yeast samples carrying different truncation forms of PALD were lysed for protein extraction. Protein samples were loaded and separated on 8% SDS-PAGE followed by Western blot analysis. The red squares are the estimated size of the specified PALD-HA. Lower panel: Coomassie blue staining was used as loading control. N: negative control, empty vector; AB: PALD-AB; FL: PALD-FL; 1 to 10: Del-1 to Del-10.

(A)

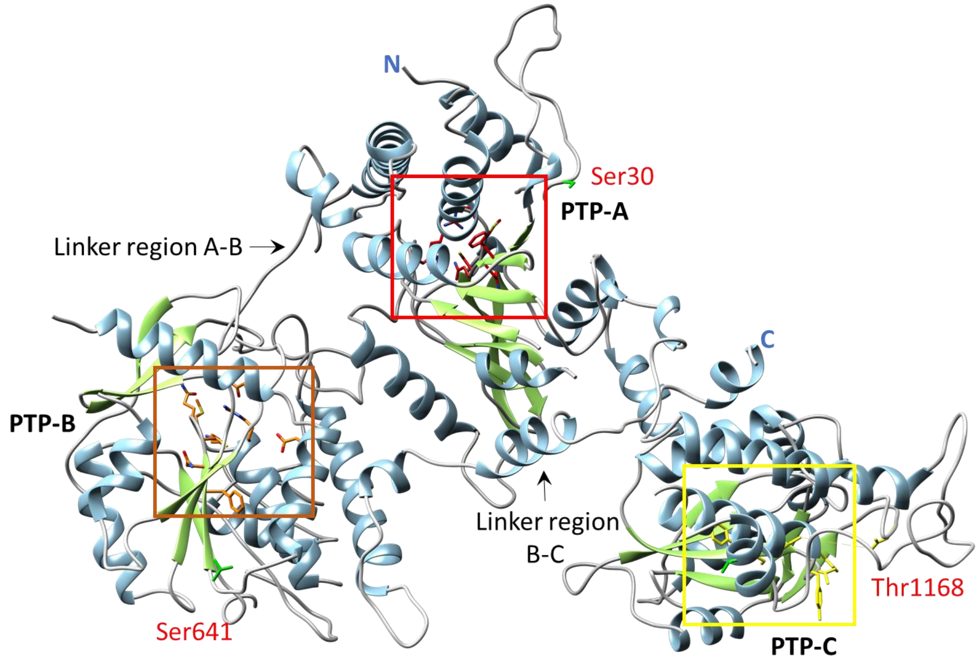

(B)

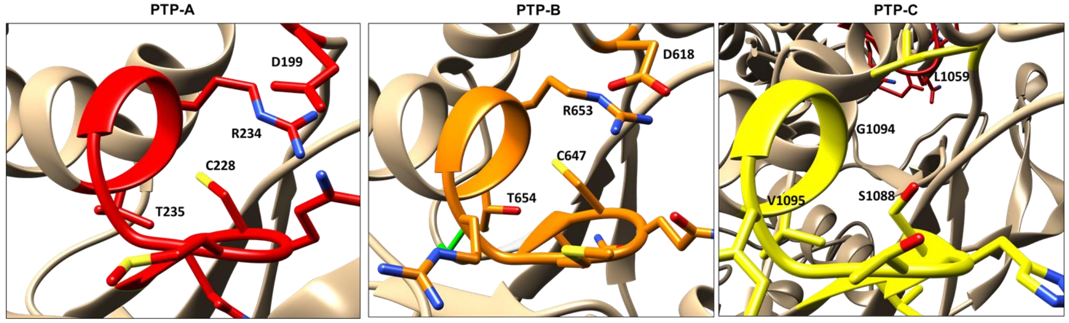

(C)

Peptides identified in MS	H_2O_1h	RaxX-sY_1h
**S**^{30}DHFPGCQNK	4	2
S^{30p}DHFPGCQNK	2	2
D**T**^{641}AFVFN**C**QMGR	0	0
D***T***^{641p}AFVFN**C**QMGR	1	1
EEVD**T**^{1168}VIDR	6	6
KEEVD**T**^{1168}VIDR	2	2
CKEEVD***T***^{1168p}VIDR	1	0

Supplemental Figure 8. Structural homology model and phosphorylated amino acid residues of PALD (A) Structural prediction of PALD crystal structure was performed using RaptorX homology modeling software (Template 4WU3). PTP domains with active site residues shown are boxed, three phosphorylated residues (Ser^{30}, Thr^{641}, and Thr^{1168} sidechains in green, labels in red)

and putative linker regions. **(B)** Model of the active site of PTP-A, B and C. PTP-A and PTP-B possess a catalytic cysteine (C228, 647), arginine (R234, 653) threonine (T235, 654) on the p-loop, and aspartate (D199, 618) on the general acid loop, while these residues are absent in PTP-C. **(C)** Three phosphopeptides were detected on HA-PALD via mass spectrometry. Three amino acids (S30, T641, and T1168) of PALD are phosphorylated *in vivo* in the absence of RaxX21-sY treatment. No significant changes were detected after RaxX21-sY peptide treatment as compared with the water control.

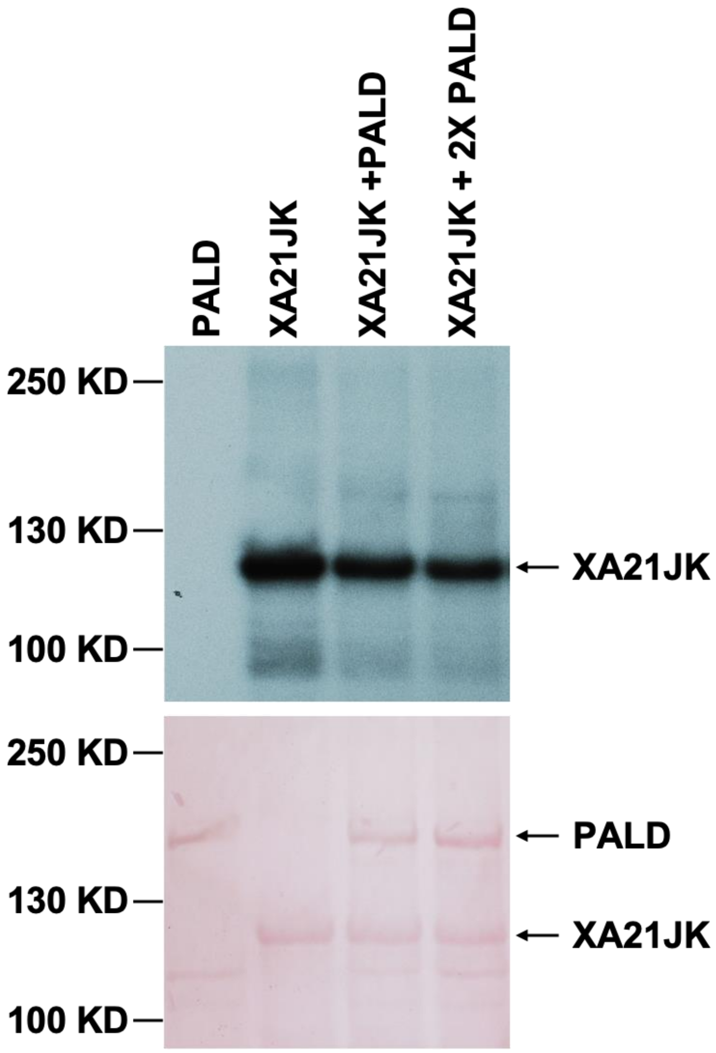

Supplemental Figure 9. XA21 does not phosphorylate PALD *in vitro*.

This *in vitro* kinase assay was performed by incubating recombinant His-Nus-XA21 in the presence or absence of His-PALD using [^{32}P]-γ-ATP. Proteins were separated by SDS-PAGE and analyzed by autoradiography. Lower panel, ponceau staining of the same PVDF membrane shown in upper panel as a protein loading control. This experiment was repeated twice with similar results.

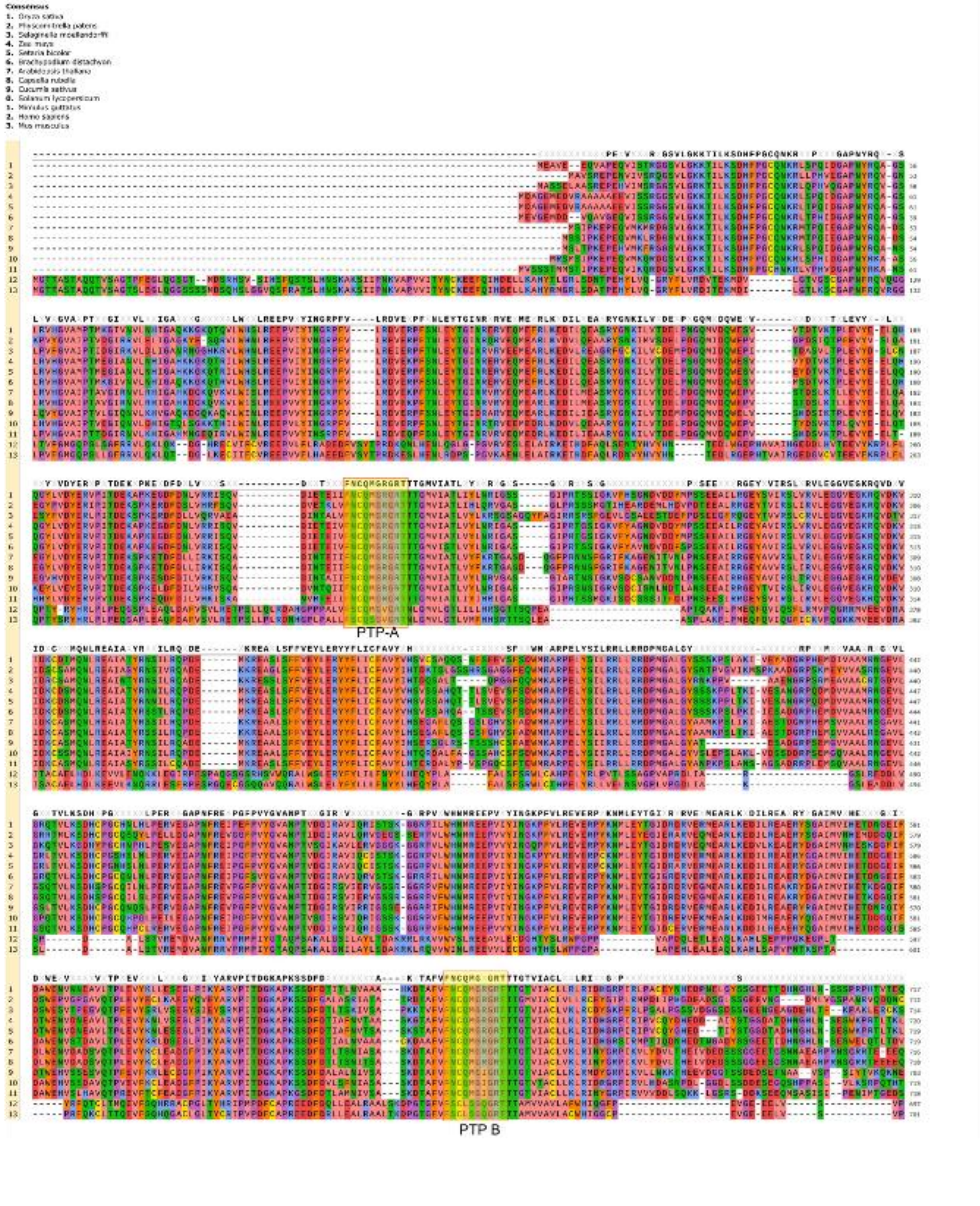
Consensus
1. Oryza sativa
2. Physcomitrella patens
3. Selaginella moellendorffii
4. Zea mays
5. Setaria bicolor
6. Brachypodium distachyon
7. Arabidopsis thaliana
8. Capsella rubella
9. Cucumis sativus
0. Solanum lycopersicum
1. Mimulus guttatus
2. Homo sapiens
3. Mus musculus
PTP-A
PTP B

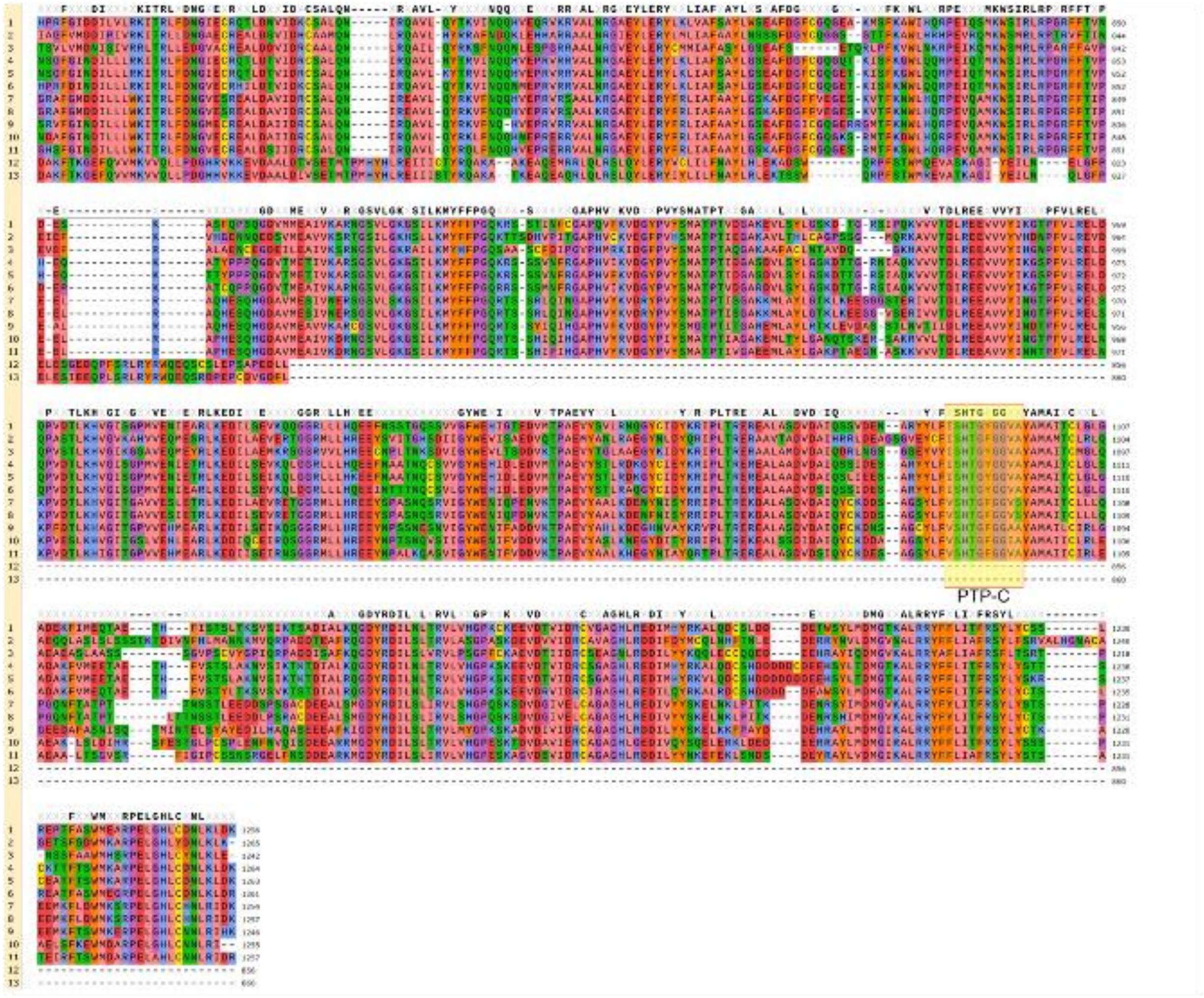

Supplemental Figure 10. Sequence alignment of select PALD proteins from plant and animals

Supplemental information on HA-PALD lines:

Among the 38 Ubi:HA-PALD/sxi2 T0 transgenic plants, 13 plants (line-#3, #6, #14b, #15b, #17, #25, #26, #27, #29, #30, #31, #32, and #35) displayed resistance to *Xoo* in at least one rice leaf, while all the other lines were susceptible (Supp. Figure 2). We harvested the seeds from the resistant tillers of the 13 plants and repeated the inoculation in the derived T1 population. We found that progeny of *Ubi:HA-PALD/sxi2*-#3, #25, and #26 are the only lines that displayed resistant phenotypes similar to the *XA21* control plants (Figure 2B). We then assessed their *PALD* RNA levels by reverse transcription quantitative PCR (RT-qPCR) analysis (Supp. Figure 3) and their protein levels by immunoblotting (Figure 2C) on the T0 populations. The RT-qPCR results showed that *PALD* transcript levels in the ten Ubi:HA-PALD/sxi2 transgenic lines (from line #1 to #10) are at least 2-fold higher than XA21 plants, suggesting that the *PALD* gene is overexpressed in these transgenic plants (Supp. Figure 3). However, immunoblot results showed that Ubi:HA-PALD/sxi2-#3, -#25, and -#26, the only resistant lines, express easily detectable levels of the HA-PALD fusion protein among all transgenic lines, whereas the remaining 35 Ubi:HA-PALD/sxi2 lines express lower or undetectable levels of the PALD protein and are susceptible to *Xoo* strain PXO99 (Figure 2B, 2C, and 2D). These results show that detectable HA-PALD protein is correlated with the *Xoo* resistant phenotype, suggesting that the PALD protein is required for *XA21*-mediated immunity.

Supplemental table S1. Primers used in this study.

Gene	Locus ID	Use	Forward primer	Reverse Primer
Actin	LOC_Os03g50885	qRT-PCR	ACAGGTATTGTGTTGGACTCTGG	AGTAACCACGCTCCGTCAGG
PALD	LOC_Os03g38970	qRT-PCR	ATCGCCAGGCGGGTTCTTTA	CGGCTCCTCGCGAAGACTAT
UBQ	LOC_Os06g46770	qRT-PCR	GTGGCCAGTAAGTCCTCAGC	ACAATGAAACGGGACACGAC
PALD	LOC_Os03g38970	Genotyping (primer 1)	AAGCGGAACGCTATAGTGGG	AAGGTGACCATTGTGATCCGTTGTT
	LOC_Os08g29590	Genotyping (Primer 2)	CACCGAATTCATGAGGATGGACAGGAATGAGGA	GTCAAGGCATGAGCGGTGAACA
	LOC_Os09g20830	Genotyping (Primer 3)	CTCTCGCTCGTGTTCGTCCTC	AGCTCCACACCTCTCTTGACCA
